# Supplementary material for: Rising prevalence of parent-reported learning disabilities among U.S. children and adolescents aged 6–17 years: NSCH, 2016–2023
Source: PLoS One. 2025 Oct 22;20(10):e0333850. doi: 10.1371/journal.pone.0333850 (PMC12543174; doi:10.1371/journal.pone.0333850)
Supplement: Attachment — Submitted filename: Comments on PONE-D-24-50967.pdf [file pone.0333850.s001.pdf]

Comments on PONE-D-24-50967

I commend the author team for their efforts to learn from an existing data set for the sake of research on the epidemiology of self-reported learning disabilities.

I may eventually speak out in favor of the manuscript being accepted for publication in *PLOS*. However, my final determination depends on how the authors address the manuscript's current weaknesses.

In sum, I have the following impression of the manuscript:

- It addresses a meaningful scientific question and takes a reasonable approach to learning from an existing data set.
- The manuscript is okay, but not yet good or excellent with regard to the scientific claims and arguments it makes. It is not yet good enough for publication in a scientific journal.
- The scientific writing (including mathematical and numerical copy) is very problematic and requires much more attention to detail and consistency. *Much hard work is needed here.*
- The English is generally understandable but is often idiomatically and grammatically incorrect. While I think it is unfair to penalize scholars whose native language happens to not be English, the standard of language use is below what I would find acceptable for a leading international peer-reviewed scientific journal. In the spirit of international collaboration, I have taken the time to describe in detail many of the language problems in need of redress. For these, if in line with PLOS guidelines, I would encourage the authors to work with a high-quality AI trained on English data. In my view, that is an unproblematic use of AI in the context of scientific writing. The authors can even 'feed' my feedback to an AI and ask for language coaching.

Finally, I have a point for the author team and the editors: I am not an expert on learning disabilities. I have published on a niche area within special education, namely on gifted education and talent development. In my view, the manuscript must also be reviewed and approved by an expert who has published on learning disabilities.

I wish the author team all the best for their work on revising the manuscript, and I am excited to see how it develops. I will only speak out in favor of publishing the manuscript in *PLOS* if the authors' next version thoroughly and thoughtfully addresses *all the points* I have raised (i.e., throughout the entire manuscript including in all tables and figures)—and with attention to detail. My expectation is, of course, not that the authors must accept and implement all of my feedback. I expect to see my feedback implemented or to receive a precise explanation about any points on which the authors disagree or see that I misunderstood.

Here are my specific points:

1. Always use en-dashes (–), never hyphens (-), for all manner of numerical ranges, including all of the numerical ranges in all of the tables. Please change this throughout the entire manuscript, including in all of the tables and figures.
2. to derive deeper understanding 🚸 to derive a deeper understanding
3. adopted the redesigned surveys, 🚸 adopted redesigned surveys,

4. The NSCH is a cross-sectional survey was conducted 🚸 Ungrammatical, please check your wording.
5. Data Source: What level of confidence do you have about just how representative the sample is of the US population? In the Introduction, you mentioned that the imputation and weighting were revised in 2024 by race and ethnicity. That suggests that care is being taken to create a helpful sample. However, the nature of the sample is so crucial for your study that I would appreciate a transparent discussion of the quality of the data. Perhaps you could consider having a subsection on the quality of the data (or something like that). Also, don't worry about 'selling' your study by overstating the quality of the data. It goes without saying that there are really no ideal samples. However, providing a critical description of your data source would add to the quality of your study by helping future researchers to build more effectively on your work. You write, for example, the following: "From 2016 to 2022, the weighted Topical Completion Rate ranged from 30.6% to 30.9%, the weighted Interview Completion Rate ranged from 69.7% to 78.5% and the weighted Overall Response Rate ranged from 37.4% to 39.1%." Explain and interpret this for your readers. With your topic, I expect that you can speak to what these numbers tell us about the quality of the data.
6. Double check your manuscript for non-typographical apostrophes (') and non-typographical quotation marks ("…") and replace these with their proper typographical forms (' 🚸 ’ and "…" 🚸 “…”) to improve the quality of your published article. *PLOS* will *not* fix these for you. They will publish what you submit 1:1.
7. told them that their child had "Learning Disability" 🚸 told them that their child had a "Learning Disability"
8. Because learning disabilities are not diagnosed until children enter school, 🚸 This sounds plausible. However, it would be better to provide a source to substantiate your claim (e.g., a leading reference work on the matter).
9. 187805 children 🚸 Always use US scientific conventions for chunking large numbers. Here, for example: 187,805 children. I expect to see this revision throughout the entire manuscript, for all numbers greater than 999, including in the tables and figures.
10. <6 years (n=90006 🚸 Review your entire manuscript for best practices for writing technical/mathematical copy. Here, for example: space after the "<" is missing; n should be italicized (*n*); spaces are needed around the equal sign; write 90,006, not 90006. I am not going to provide feedback on this for the rest of the article. Please thoroughly review and improve your adherence to international standards of scientific writing in how you express numbers etc. The current standard is too low and would reflect poorly on the quality of your work.
11. Information on sex, race/ethnicity, family income and highest education 🚸 Information on sex, race/ethnicity, family income, and highest education 🚸 Learn how to use the serial comma for enumerations with 'and' or 'or' and apply that rule consistently throughout your entire manuscript. I will only note this one case, so you know what I am talking about. There are many such cases that need to be attended to. Ask an AI trained on US (not British!) language data to coach you on proper use of the "serial comma" before 'and' and 'or'
12. the 1997 Office of Management and Budget Standards. 🚸 Reference is missing. Add.
13. Federal poverty level (FPL), 🚸 federal poverty level (FPL), 🚸 'Federal' should not be capitalized.

14. 100-199%FPL, 200-399%FPL, and 400%FPL or above 🚸 Spaces are missing. Cut and paste error! Also note here the failure to use en-dashes for numeric ranges (many such instances need to be fixed).
15. Logistic regression model was used 🚸 A logistic regression model was used 🚸 You are very frequently avoiding using definite and indefinite articles. While this is acceptable to a certain point in scientific writing, it reads awkwardly in your manuscript. If you are not sure about how to use definite and indefinite articles properly, ask an AI trained on English language data to help you with that.
16. members**.** 🚸 members. The period here is set in boldface. That must be by accident. *PLOS* will not correct this sort of typographical error for you!
17. The 2 sided statistical significance level was set at α=0.05. 🚸 The 2-sided statistical significance level was set at α = 0.05. 🚸 Please make sure to improve your following of conventions related to all mathematical copy in your manuscript. This is just one example. There are many more, also in the tables and figures.
18. And a methods question about your use of a two-sided statistical significance level: You chose this option because you are making no directional assumptions about the hypotheses you are testing? I would have expected a brief remark explaining why you made the choice you did.
19. This study included 187805 children and adolescents aged 6-17 years (97123 boys [51.71%], 90682 girls [48.29%], 24184 Hispanic [12.88%], 127277 non-Hispanic white [67.77%], 12491 non-Hispanic black [6.65%], 23853 other race/ethnicity [12.70%]) in 2016-2022. 🚸 I suggest using a semicolon in your parenthetical remark to break the groups up into meaningful subsets that sum up to the total. In other words: This study included 187,805 children and adolescents aged 6–17 years (97,123 boys [51.71%] and 90,682 girls [48.29%]; 24,184 Hispanic [12.88%], 127,277 non-Hispanic white [67.77%], 12,491 non-Hispanic Black [6.65%], and 23,853 other race/ethnicity [12.70%] individuals) for the period 2016–2022.
20. have ever been diagnosed with LD 🚸 have ever been diagnosed with an LD [see my previous comment on this problem; there are MANY such instances in need of improvement]
21. Utah (6.07% of ever-diagnosed LD and 5.70% of current LD) (Figure 2) (Figure 3). 🚸 First, please re-check the *PLOS* rules for the wordings to be used for things like 'Figure' and 'Table.' You are supposed to write 'Fig 1' and 'Table 1,' for example. That always strikes me as strange, but that is the journal rule (at least as of last year). Second, avoid having a series of parentheses following each other. Rather: Utah (6.07% of ever-diagnosed LD and 5.70% of current LD; see Fig 2–3).
22. We found that age, sex, race/ethnicity, family income and highest education of family members were significant differences in both Ever LD and Current LD. 🚸 We found that age, sex, race/ethnicity, family income, and highest education of family members were significant differences in both the Ever LD and Current LD groups. 🚸 Please note in my revision suggestion not only the main change (towards the end) but also the addition of the serial comma before 'and highest.' There are many such instances in your manuscript in which the serial comma is missing. Make sure to fix those. See my previous remark for a suggestion of how do work on this.
23. Ever LD, 9.70%, 95%CI, 9.31-10.09; 🚸 Ever LD, 9.70%, 95% CI, 9.31–10.09; 🚸 Many such corrections need to be made. In particular, it makes no sense to not have a space before 'CI'
24. In the sex group, ... 🚸 With regard to children's sex, ... [Don't write 'sex group']
25. The weighted prevalence of Ever LD and Current LD in US children and adolescents aged 6 to 17 years were 🚸 Consider putting the data that follow into a table. That

is really hard for your readers to actually read! Also, it is much more important to explain what you found and WHY the trend is important to take not of. At the end of the paragraph, remember to apply my feedback from above about not having a series of parentheses: (P for trend < .01) (Table 2) (Table 3) (Figure 4). 🚸 That is not a conventional way of expressing that information.

26. (P for trend < .01) 🚸 Why an upper-case *p* here? You mean a regular *p* value, no? Remember also to take care to follow conventions on which letters need to be italicized. Also, why do you change the significance level for this? Please explain. Perhaps you are being more cautious to avoid reporting a spurious trend?
27. 200 %FPL 🚸 200% FPL 🚸 Check and apply the rules for writing percentages in scientific copy and apply them consistently throughout your entire manuscript.
28. We found a significant average annual change in aged 12-17 years, female, non-Hispanic white, a college or higher education, and family income of 200 %FPL or above in the prevalence of both Ever LD and Current LD 🚸 This statement needs to be reworded to make it easier to read. For example: For the prevalence of Ever LD and Current LD, we found significant average annual changes for the following demographic categories: female, non-Hispanic white, a college or higher education, and family income of 200% FPL or above. 🚸 I cannot be sure my wording is factually correct. That is your job. However, I want to illustrate what is probably a better wording. You could ask an AI trained on English data to help you improve such wordings.
29. In a nationally representative population from the NSCH, we identified a weighted prevalence of ever-diagnosed LD at 8.80% and current-diagnosed LD at 8.20% among US children aged 6-17 years in 2016-2022. 🚸 In a nationally representative population from the NSCH, we identified a weighted prevalence of ever-diagnosed LD at 8.80% and current-diagnosed LD at 8.20% among US children who were aged 6–17 years in the period 2016–2022.
30. in aged 12-17 years, 🚸 I literally do not understand what you mean with this wording (here and earlier in the manuscript). Ask an AI why this is hard for readers to understand what you mean. I am sure you mean something meaningful. However, there is probably a language barrier leading to a wording in English that is confusing. The rest of the phrase makes sense but should be reworded (see above).
31. A study analyzing the association between screen time and developmental disabilities among children aged 0-17 years using NSCH data from 2018-2020, noted a total LD 🚸 Remove the comma here. It is incorrect and confusing. Ask an AI to explain to you WHY the comma is wrong.
32. another research conducted by Zablotsky, 🚸 another study conducted by Zablotsky, 🚸 It is grammatically incorrect to write “another research” because “research” is a mass noun (i.e., you cannot count it; like the word ‘milk,’ for example). You cannot say “a” research. Here, too, ask an AI to help you understand what I mean. Writing “Other research” is fine, however, because “other” does not quantify what follows the way “another” does.
33. Moreover, it is a lifelong condition 🚸 I highly doubt that the state of the art within theories of learning disabilities agree that learning disabilities are always lifelong conditions. Also, writing “it is a lifelong condition” is weak both from a writing and a thinking standpoint. What EXACTLY do you mean here by “it”? Please provide a more nuanced statement and align it very carefully with leading textbooks / technical literature on learning disabilities. Perhaps learning disabilities are “often” lifelong conditions? At any rate, be more nuanced and follow recent cutting-edge theory in the field.

34. Moreover, it is a lifelong condition and older children may have a higher prevalence rate and increasing trend due to longer exposure to the possibility of evaluation and diagnosis.7 There are several problems with this sentence: First, as noted, the claim about "it" being a "lifelong condition" seems implausible and simplistic. Second, your sentence makes THREE major claims, all connected backwards with "Moreover." That is not a good writing style, and it is not argumentatively persuasive to pack all this stuff into one sentence and only have one reference at the end of the sentence. A better approach: Break the claims up. Carefully explain each claim and offer literature support for each claim separately. In other words: First claim: About the long-term nature of (many) learning disabilities [add supporting literature]. Second claim: higher prevalences are being reported for older children [add supporting literature]. Third claim: The higher prevalences being observed for older children may be resulting from ... [explain that idea in detail and then add supporting literature].
35. Learning disabilities are difficult to diagnose, because the age at identification depends not only on the type and severity of the learning disabilities, but also on whether there are associated deficits.25 ... This is a very important paragraph for your discussion. What is missing is some sort of advanced organizer after the introductory part of the paragraph explaining the nature of the different observations that then constitute your analysis in the paragraph. After the first two sentences (as of "Nearly all studies ..."), you then discuss gender differences, then ethnic group differences, then SES differences. Prepare the reader for THAT structure before 'diving into' those areas. Do you understand what I mean? If you are not sure, show the paragraph and my comment to an AI and ask for help on how to improve the organization of your thoughts here. You might even consider breaking the paragraph up: first paragraph: Main claim and the advanced organizer + second paragraph about the gender differences + third paragraph about ethnic differences + fourth paragraph about the SES differences.
36. Autism Spectrum Disorder (ASD) Check, for example, the APA7 guidelines on writing the names of conditions, disorders, and the like. I'm pretty sure you write them lowercase. Thus: autism spectrum disorder (ASD)
37. Although the pathogenesis of learning disabilities is currently unclear, I'm skeptical that your blanket statement is accurate. Are you sure this is what state-of-the-art handbooks and the like are currently saying about learning disabilities? I would imagine that the pathogenesis of learning disabilities is not fully understood, but that much is known about at least some of the complex etiological factors that have been implicated in contributing to specific learning disabilities. The term 'learning disabilities' covers a huge range of conditions that themselves have all sorts of definitions. Please re-read a few introductory handbook chapters in the field and reconsider your statement to make sure you are being nuanced in how you make this point.
38. Certain heritable disorders even approach 100% penetrance, What does "penetrance" mean here? Are you sure that is the right word? I'm not sure that is actually a word. Do you mean "penetration"? But if yes, what would that mean?
39. Dietary behaviors have also been proposed to play a role in the increases in LD, exemplified by a study that found a high intake of sweetened desserts, fried food, and salt was associated with more learning disability.50 Hm, are you sure this is a strong study? Without knowing the study, my hunch would be that we are talking about comorbidities and that it is really hard to get at causality here. If you cite the study, make sure it is a strong study that actually makes progress towards

substantiating a causal link. Is the study an RCT? Was it at least longitudinal? Did they at least use propensity-score matching? Otherwise, I would imagine that parents who allow their children to consume excessive amounts of unhealthy food are also engaging a host of other behaviors that are harmful for their children's development. Be cautious here. If the study is weak, consider removing or revising your claim.

40. associated with learning disability, 🚸 associated with learning disabilities, 🚸 That is not a trivial language difference in English. I know it looks like a tiny change (from singular to plural), but it is very important that you become aware of these sorts of usage conventions. If you are not sure whether your wording is idiomatic, ask an AI trained on English to help you check your writing. Feel free to share my feedback with an AI so the AI can help you with your language in the manuscript.
41. Many proven or potential environmental biological factors 🚸 Many substantiated or implicated environmental-biological factors 🚸 I would avoid "proven" in this sort of research, because it is generally not possible to truly 'prove' these sorts of relationships. At best, we find stronger/better evidence.
42. showed that children in the highest quartile of urinary cadmium 🚸 "urinary cadmium" what? You probably mean in the highest quartile of cadmium levels in urine samples. If you are taking the term "urinary cadmium" from an article on that topic, say, then the authors have probably established with what they mean by "urinary cadmium" before they start using that shortened form. Check the literature you are citing and be more thorough in your wording here.
43. had significantly higher odds of LD 🚸 had significantly higher odds of having a self-reported LD
44. Research has confirmed that learning disabilities are lifelong;9 🚸 This again sounds very simplistic. I highly doubt that "research" has literally "confirmed" that (all) "learning disabilities"—which is a giant blanket term covering probably hundreds if not thousands of researcher-defined conditions—are "lifelong." Follow my feedback from above on this point. This is an essential point that must be improved in your writing. Otherwise, your thinking is not nuanced enough for a serious scientific publication. To make sure you are not discouraged by my strong feedback here: Your larger point, which I think is that learning disabilities have long-term, potentially even lifelong consequences for individuals and, therefore, need to be given much attention, is a good and strong point that you definitely can and should make in your article 😊
45. with specific learning disability amounted 🚸 with a specific learning disability amounted 🚸 Here, too: This might seem like a tiny wording change. However, it is a very important chance for learning to write polished English scientific prose. Ask an AI trained on English to help you learn how to catch and fix these sorts of wording issues.
46. In the US, it's reported that over half of the students receiving special education services do so due to learning disabilities..11 🚸 In the US, over half of students receiving special education services receive them due to learning disabilities.11 🚸 Avoid using contractions in formal scientific papers (it's 🚸 it is); it goes without saying that this has been reported; moreover, I do wonder about the point you are making conceptually: What does that statistic tell us since I would imagine that access to special educational services normally REQUIRES some sort of diagnosis of a learning disability? Consider addressing that point. My guess is that it is explained in the study you cite. If I am missing your point, then explain your point in the manuscript for thoroughly.

47. This study has several limitations. First, the cross-sectional design of the NSCH does not permit the identification of how specific factors may influence the diagnosis of LD. Second, the reliance on parental reports for the diagnosis of LD could potentially invite inaccuracies due to misreporting and recall bias. This is your entire discussion of limitations? I find it rather superficial and unconvincing. The authors need to take a serious look at the sample and their method of analyzing the sample and think critically about important limitations and discuss these carefully. I already noted one limitation above about the same. Moreover, you are working with self-reported learning disabilities. Furthermore, you have an extremely undifferentiated operationalization that literally includes all manner of 'learning disabilities.' What's more, you are publishing your research for an international audience but focusing on internal US data. What does that limitation mean for the generalization of your findings beyond the United States? This is a big problem in research in the social sciences including psychology; see, for example, the work on the WEIRD bias by Joseph Henrich.

48. I am *not* an expert on learning disabilities, but you authors are, so please put much more effort into creating a well-informed, sincere, and deep discussion of limitations. With a good discussion of limitations, you will greatly improve the scientific value of your work, because science is about building constructively on existing studies. If you fail to provide a thorough and transparent discussion of your study limitations, you are essentially blinding researchers with regard to your work, making it harder for them to build on your work. I have a problem with that. Also, as your limitations section is currently written, researchers will be skeptical when they read your article and thus less likely to cite you. In other words, it is also a matter of establishing trust with your readers by having a detailed and brave limitations section. To be clear: I will not reject a study for being open and thorough about limitations. On the contrary, a deep discussion of limitations is a hallmark of scientific thinking.

49. Conclusions: The conclusion is lacking in depth. I agree that you should keep your conclusion focused on concrete key findings and implications and that, therefore, all manner of 'fluff' is to be avoided. However, I think you can do much better here.

Finally, I have one suggestion that I would like to offer as food for thought, as we say in English—perhaps similar in spirit to the idea of 抛砖引玉:

The author team is located in China, at Chinese universities. From my perspective, China is a key player and a leader for all of humanity in the twenty-first century. It is home to a significant portion of the world's population and shares deep cultural affinities with other East Asian societies. With that in mind, the authors might consider adding a paragraph in the Discussion or Conclusion section reflecting on the relevance of their US-centric findings for the epidemiology of learning disabilities in China—and, more broadly, in collectivist or Confucian-tradition cultures. Concepts from Chinese educational and societal traditions—such as moral education (德育), teaching according to aptitude (因材施教), inclusive education (融合教育), and collectivism (集体主义)—may offer helpful points of reference. The authors might also reflect on how learning disorders are labeled and ascribed to individuals, particularly in light of the Confucian principle of the rectification of names (正名), which underscores the ethical importance of ensuring that classifications align with actual roles and behaviors. The history of special education in the United States—and the framing of learning disabilities more broadly—is deeply embedded in American cultural norms and assumptions. For comparative context, the authors may wish to consult the work of Harold

W. Stevenson and those who have continued his line of research over the past two decades. Research originating in the US is, of course, and will remain relevant to international scholarship. In a globally connected world, many questions of human functioning can be fruitfully explored using data from diverse national contexts. Still, findings from any one culture must be interpreted with cultural awareness and thoughtful regard for how such conclusions are to be meaningfully mapped onto other traditions and systems of thought. While my final vote on the publication of your manuscript in *PLOS* (still pending) depends on the extent to which you address the concerns I raised above, I would like to make clear that this final suggestion is *not* part of that evaluation. It is entirely up to the authors whether they choose to include a remark on the Chinese cultural context. My verdict on the manuscript does not depend on it.
