# Supplementary material for: Rising prevalence of parent-reported learning disabilities among U.S. children and adolescents aged 6–17 years: NSCH, 2016–2023
Source: PLoS One. 2025 Oct 22;20(10):e0333850. doi: 10.1371/journal.pone.0333850 (PMC12543174; doi:10.1371/journal.pone.0333850)
Supplement: Attachment — Submitted filename: Comments on PONE-D-24-50967_R1.pdf [file pone.0333850.s003.pdf]

Comments on PONE-D-24-50967, Version 2

The authors have made vast improvements to the manuscript, both on conceptual aspects and on applying conventions of scientific writing, and I congratulate them on that. I appreciate very much the time and effort they invested, also in the many of my points that are related to secondary aspects of the scientific process (communication conventions).

Most—but not all—of the technical matters I noted in my first set of comments have been successfully addressed. Before I can agree that the manuscript should be published, the remaining points noted below need to be addressed. Please address (a) the remaining issues from my feedback of 29 April (described here in section 1) and (b) the small additional problems I noted when reading your revised manuscript (described here in section 2).

1. Assessment of Extent to which the Authors Addressed My Points of 29 April 2025 and Issues that Are Not Fully Addressed

No.	Specific Point	Addressed?	Approved for Publication
1	Always use en-dashes (–), never hyphens (-), for all manner of numerical ranges, including all of the numerical ranges in all of the tables. Please change this throughout the entire manuscript, including in all of the tables and figures.	Much improved, but not fully revised. Please fix all such remaining problems:<br>• disorders——such as → disorders—such as<br>• difficulties——not clinically → difficulties—not clinically<br>• Rao-Scott chi-square tests → Rao–Scott chi-square tests [Between Rao and Scott, you need an en-dash (–), not a hyphen (-) and also not an em-dash (—). Between “chi” and “square” you did it correctly with a regular hyphen (-).]<br>• 2 sided statistical significance level → 2-sided statistical significance level [”2 sided” needs to be hyphenated because it is being used as an adjectival compound; for *some* compound adjectives, you need to use hyphens; the rules are a bit complicated and there are many edge cases; here, it is clear that you need the hyphen]<br>• Fig 1. Weighted Prevalence Estimates of Parent-Reported Ever Learning Disability (LD) among Children Aged 6–17 Years by State ——United States, 2016–2023 → The mistakes here are that you (a) have an extra space between “State” and the em-dash and (b) have two em-dashes (——), not one (—). However, from a stylistic standpoint, I would advise you to not use an em-dash at this point. Instead, perhaps: Fig 1. Weighted Prevalence Estimates of Parent-Reported Ever Learning Disability (LD) among Children Aged 6–17 Years by State (United States, 2016–2023)<br>• I find the same errors (and suggest the same possible stylistic change) in Fig 2<br>• Triple-check the tables. I didn’t check them carefully.	No

No.	Specific Point	Addressed?	Approved for Publication
2	to derive deeper understanding to derive a deeper understanding	Yes, good!	Yes
3	adopted the redesigned surveys, adopted redesigned surveys,	Yes, good!	Yes
4	The NSCH is a cross-sectional survey was conducted Ungrammatical, please check your wording.	Yes, good!	Yes

No.	Specific Point	Addressed?	Approved for Publication
5	Data Source: What level of confidence do you have about just how representative the sample is of the US population? In the Introduction, you mentioned that the imputation and weighting were revised in 2024 by race and ethnicity. That suggests that care is being taken to create a helpful sample. However, the nature of the sample is so crucial for your study that I would appreciate a transparent discussion of the quality of the data. Perhaps you could consider having a subsection on the quality of the data (or something like that). Also, don't worry about 'selling' your study by overstating the quality of the data. It goes without saying that there are really no ideal samples. However, providing a critical description of your data source would add to the quality of your study by helping future researchers to build more effectively on your work. You write, for example, the following: "From 2016 to 2022, the weighted Topical Completion Rate ranged from 30.6% to 30.9%, the weighted Interview Completion Rate ranged from 69.7% to 78.5% and the weighted Overall Response Rate ranged from 37.4% to 39.1%." Explain and interpret this for your readers. With your topic, I expect that you can speak to what these numbers tell us about the quality of the data.	Yes, sort of. For future rebuttal letters: Please do not paraphrase reviewer feedback when addressing reviewer points. This makes it more difficult for me to check whether you have adequately addressed my concern and is generally not good practice for a rebuttal letter. I don't have extra time to think through whether your paraphrase of my words captures all of my concerns in the manner I meant them.	Yes

No.	Specific Point	Addressed?	Approved for Publication
6	Double check your manuscript for non-typographical apostrophes (') and non-typographical quotation marks ("…") and replace these with their proper typographical forms (' → ’ and "…" → “…”) to improve the quality of your published article. PLOS will not fix these for you. They will publish what you submit 1:1.	Much improved, but not fully revised. Please fix:<br>• a family's level of education → a family’s level of education<br>• If you are using AI to give you language feedback, be very carefully when pasting in revised wordings suggested by an AI. Unless you instruct the AI to do so, it may very likely not use proper typographical quotation marks, apostrophes, and the like.	No
7	told them that their child had “Learning Disability” → told them that their child had a “Learning Disability”	Yes, good!	Yes
8	Because learning disabilities are not diagnosed until children enter school, → This sounds plausible. However, it would be better to provide a source to substantiate your claim (e.g., a leading reference work on the matter).	Yes, and excellent changes and explanation. Thank you!	Yes
9	187805 children → Always use US scientific conventions for chunking large numbers. Here, for example: 187,805 children. I expect to see this revision throughout the entire manuscript, for all numbers greater than 999, including in the tables and figures.	Yes, good!	Yes

No.	Specific Point	Addressed?	Approved for Publication
10	<6 years (n=90006 Review your entire manuscript for best practices for writing technical/mathematical copy. Here, for example: space after the "<" is missing; n should be italicized (*n*); spaces are needed around the equal sign; write 90,006, not 90006. I am not going to provide feedback on this for the rest of the article. Please thoroughly review and improve your adherence to international standards of scientific writing in how you express numbers etc. The current standard is too low and would reflect poorly on the quality of your work.	Much improved, but not fully revised. Please fix all such remaining problems. For example:<br>• lasting ≥6 months; → lasting ≥ 6 months;<br>• children <6 years → children < 6 years<br>• and ≥400% FPL → and ≥ 400% FPL<br>• *p* values in all three tables: for example: <0.001 → < 0.001<br>• *P* for trend <0.001 → *P* for trend < 0.001 [multiple instances]<br>• ≥200% FPL. → ≥ 200% FPL.<br>• (<1.00: $P = 0.777$; … → (< 1.00: $P = 0.777$; …<br>• 0.001; ≥4.00: $P < 0.001$). → There is a space missing after "≥". Also, is a *P* missing there, too?<br>• where >95% → where > 95%<br>• I'm pretty sure there are a few more such instances. Check the various mathematical relationship signs to make sure you don't miss any needed corrections of this sort.	No
11	Information on sex, race/ethnicity, family income and highest education Information on sex, race/ethnicity, family income, and highest education Learn how to use the serial comma for enumerations with 'and' or 'or' and apply that rule consistently throughout your entire manuscript. I will only note this one case, so you know what I am talking about. There are many such cases that need to be attended to. Ask an AI trained on US (not British!) language data to coach you on proper use of the "serial comma" before 'and' and 'or'	Much improved, but not fully revised. Please fix all such remaining problems:<br>• were asked if a doctor, other health care provider or educator had → were asked whether a doctor, other healthcare provider, or educator had [the change of "if" to "whether" and health care to "healthcare" are bonus corrections]<br>• on ever diagnosis information ($n = 803$), current diagnosis information ($n = 256$) and highest parental education ($n = 875$). → on ever-diagnosis information ($n = 803$), current-diagnosis information ($n = 256$), and highest parental education ($n = 875$). [the two added hyphens are bonus stylistic suggestions (not mandatory) for recommended hyphenated adjectival compounds]<br>• prevalence on families, schools and the healthcare system → prevalence on families, schools, and the healthcare system	No
12	the 1997 Office of Management and Budget Standards. Reference is missing. Add.	Yes, good!	Yes

No.	Specific Point	Addressed?	Approved for Publication
13	Federal poverty level (FPL), federal poverty level (FPL), 'Federal' should not be capitalized.	Yes, good!	Yes
14	100-199%FPL, 200-399%FPL, and 400%FPL or above Spaces are missing. Cut and paste error! Also note here the failure to use en-dashes for numeric ranges (many such instances need to be fixed).	Yes, good!	Yes
15	Logistic regression model was used A logistic regression model was used You are very frequently avoiding using definite and indefinite articles. While this is acceptable to a certain point in scientific writing, it reads awkwardly in your manuscript. If you are not sure about how to use definite and indefinite articles properly, ask an AI trained on English language data to help you with that.	Yes, good!	Yes
16	members**.** members. The period here is set in boldface. That must be by accident. PLOS will not correct this sort of typographical error for you!	Yes, good!	Yes
17	The 2 sided statistical significance level was set at α=0.05. The 2-sided statistical significance level was set at α = 0.05. Please make sure to improve your following of conventions related to all mathematical copy in your manuscript. This is just one example. There are many more, also in the tables and figures.	No. Something must have gone wrong either when I copied my feedback into the online review form or when you worked with it. You must write "The 2-sided statistical significance level …" (with a hyphen). The hyphen is missing is my wording as noted in your rebuttal letter, even though I know I typed a hyphen. Please fix this in the manuscript. If you are unsure what I mean, please recheck the PDF version of my feedback of 29 April:<br>17. The 2 sided statistical significance level was set at α=0.05. The 2-sided statistical significance level was set at α = 0.05. Please make sure to improve your following of conventions related to all mathematical copy in your manuscript. This is just one example. There are many more, also in the tables and figures.	No

No.	Specific Point	Addressed?	Approved for Publication
18	And a methods question about your use of a two-sided statistical significance level: You chose this option because you are making no directional assumptions about the hypotheses you are testing? I would have expected a brief remark explaining why you made the choice you did.	Yes, and thank you for the explanation!	Yes
19	This study included 187805 children and adolescents aged 6-17 years (97123 boys [51.71%], 90682 girls [48.29%], 24184 Hispanic [12.88%], 127277 non-Hispanic white [67.77%], 12491 non-Hispanic black [6.65%], 23853 other race/ethnicity [12.70%]) in 2016-2022. I suggest using a semicolon in your parenthetical remark to break the groups up into meaningful subsets that sum up to the total. In other words: This study included 187,805 children and adolescents aged 6–17 years (97,123 boys [51.71%] and 90,682 girls [48.29%]; 24,184 Hispanic [12.88%], 127,277 non-Hispanic white [67.77%], 12,491 non-Hispanic Black [6.65%], and 23,853 other race/ethnicity [12.70%] individuals) for the period 2016–2022.	Yes, fantastic!	Yes
20	have ever been diagnosed with LD have ever been diagnosed with an LD [see my previous comment on this problem; there are MANY such instances in need of improvement]	Probably addressed due to your clear addressing of the problem at the earlier comment. For future rebuttal letters: Even if it is almost obvious that you have addressed a concern, please include all points shared as feedback with exactly the same wording and in the same order as shared by the reviewer in your rebuttal letter. Skipping a wording or a point here or there makes it much more difficult for the reviewer to quickly check that you systematically addressed all of their points. For example, your number of my points no longer matches 1:1 my numbering in my original feedback letter, which complicates things.	Yes

No.	Specific Point	Addressed?	Approved for Publication
21	Utah (6.07% of ever-diagnosed LD and 5.70% of current LD) (Figure 2) (Figure 3). First, please re-check the PLOS rules for the wordings to be used for things like ‘Figure’ and ‘Table.’ You are supposed to write ‘Fig 1’ and ‘Table 1,’ for example. That always strikes me as strange, but that is the journal rule (at least as of last year). Second, avoid having a series of parentheses following each other. Rather: Utah (6.07% of ever-diagnosed LD and 5.70% of current LD; see Fig 2–3).	Not exactly, but this is okay. At the bottom of page 30, you still have adjacent sets of parentheses: The weighted prevalence of Ever LD rose from 7.86% (95% *CI*, 7.25–8.46) to 9.15% (95% *CI*, 8.55–9.75) (*P* for trend <0.001), while Current LD increased from 7.32% (95% *CI*, 6.73–7.91) to 8.66% (95% *CI*, 8.06–9.25) (*P* for trend <0.001), → I understand why you didn’t want to collapse the information in the two sets of adjacent parentheses into one set in this case. However, there are other ways of avoiding the double sets of adjacent parentheses that would also provide for correctly parsed information. Find another solution, if possible.	Yes, because this is a trivial issue and more of a style question but try to improve the solution to be more in line with best practices. The missing spaces after “<” are not approved for publication. However, I list that in another row.
22	We found that age, sex, race/ethnicity, family income and highest education of family members were significant differences in both Ever LD and Current LD. We found that age, sex, race/ethnicity, family income, and highest education of family members were significant differences in both the Ever LD and Current LD groups. Please note in my revision suggestion not only the main change (towards the end) but also the addition of the serial comma before ‘and highest.’ There are many such instances in your manuscript in which the serial comma is missing. Make sure to fix those. See my previous remark for a suggestion of how do work on this.	Much improved, but not fully revised. Please fix all such remaining problems with the missing serial comma. See the specific observations I added in the line on Point 11.	No
23	Ever LD, 9.70%, 95%CI, 9.31-10.09; Ever LD, 9.70%, 95% CI, 9.31–10.09; Many such corrections need to be made. In particular, it makes no sense to not have a space before ‘CI’	Yes, good!	Yes

No.	Specific Point	Addressed?	Approved for Publication
24	In the sex group, ... 🚸 With regard to children’s sex, ... [Don't write 'sex group']	Yes, good!	Yes
25	The weighted prevalence of Ever LD and Current LD in US children and adolescents aged 6 to 17 years were 🚸 Consider putting the data that follow into a table. That is really hard for your readers to actually read! Also, it is much more important to explain what you found and WHY the trend is important to take not of. At the end of the paragraph, remember to apply my feedback from above about not having a series of parentheses: (P for trend < .01) (Table 2) (Table 3) (Figure 4). 🚸 That is not a conventional way of expressing that information.	Yes, good!	Yes

26 (P for trend < .01) Why an upper-case *p* here? You mean a regular *p* value, no? Remember also to take care to follow conventions on which letters need to be italicized. Also, why do you change the significance level for this? Please explain. Perhaps you are being more cautious to avoid reporting a spurious trend?	I am not satisfied with your responses here.<br>• First, and most importantly: The practice is almost universal to write a lowercase *p* rather than an uppercase *P* for discussions of statistical significance. The convention of writing "*P* value" or "*P* = 0.05," for example, is outdated. One writes a capital P (but not italicized) when discussing probability theory in mathematical texts (e.g., P(A)), however that is not relevant for empirical discussions of hypotheses and the like in methods sections of research papers with empirical data. Please correct all *p* values to a lowercase *p* or provide me with an unambiguous current reference (not to an ai response) to a leading style guide for statistical copy that mandates writing a capital letter for *P* values.<br>• Second, there was a misunderstanding in how your read my comment. Only the letter *p* is to be italicized. Your second response suggests that you thought I meant that additional words (~~*P for trend*~~) are to be italicized. That is not what I meant. Indeed, as you explained, that makes no sense.<br>• Third, while one can write "*p*-value" in some conventions, APA style, for one, explicitly mandates writing "*p* value" without a hyphen. Please be aware of this, even if you choose to write "*p*-value" in line with another convention.<br><br>To support your reflection on the first point here, please take a look at my discussion of this question with Claude.ai:<br>**<br>*My Prompt:*<br><br>Are there instances in best practices in statistics and statistical copy in which you write a *p* value as a capital letter?<br><br>*Answer from Claude.ai:*<br><br>In statistical writing and reporting, the *p*-value is almost universally written with a lowercase "*p*" in standard practice. This convention is followed by major style guides including:<br><br>APA Style (American Psychological Association)<br>AMA Manual of Style (American Medical Association)<br>Journal style guides across disciplines<br>Statistical software output conventions	No

The lowercase "*p*" is used whether you're writing "$p < 0.05$", "$p = 0.032$", or "*p*-value" in running text.

However, there are a few specific instances where you might see a capital "*P*":

At the beginning of a sentence: "*P*-values less than 0.05 were considered significant"—though many style guides actually recommend rewording to avoid starting sentences with statistical symbols.

In some older statistical texts or papers: Occasionally you'll find "*P*-value" in older literature, but this has largely been standardized to lowercase.

Certain mathematical contexts: In formal probability theory, you might see "P(A)" for probability of event A, but this is different from the *p*-value concept in hypothesis testing.

Some non-English statistical traditions: Certain international or non-English statistical communities may have different conventions.

The overwhelming best practice recommendation is to use lowercase "*p*" consistently in statistical reporting. [highlighted by me to draw your attention to what I think is the most important conclusion in the response] If you're writing for publication, check the specific journal's style guide, *but lowercase will be correct in virtually all cases.*

My Follow-Up Question for Claude.ai:

Also, at least according to APA style it is "*p* value" and not, as you wrote, "*p* value," correct?

Claude.ai's Response:

You're absolutely correct! According to APA Style, it should be written as "*p* value" (with a space) and not "*p*-value" (with a hyphen).

APA Style specifically uses:

"*p* value" in running text
"$p < 0.05$" or "$p = 0.032$" when reporting the actual statistic

No.	Specific Point	Addressed?	Approved for Publication
		Thank you for the correction. The hyphenated form “*p*-value” is actually more common in some other style guides and general usage, but APA Style indeed uses the space rather than the hyphen. This is an important distinction for anyone following APA formatting requirements for academic papers or publications.<br>**	
27	200 %FPL 200% FPL Check and apply the rules for writing percentages in scientific copy and apply them consistently throughout your entire manuscript.	Probably. I cannot find your response on this point. A quick check of your manuscript shows that you did address this point, I think.	Yes
28	We found a significant average annual change in aged 12-17 years, female, non-Hispanic white, a college or higher education, and family income of 200 %FPL or above in the prevalence of both Ever LD and Current LD This statement needs to be reworded to make it easier to read. For example: For the prevalence of Ever LD and Current LD, we found significant average annual changes for the following demographic categories: female, non-Hispanic white, a college or higher education, and family income of 200% FPL or above. I cannot be sure my wording is factually correct. That is your job. However, I want to illustrate what is probably a better wording. You could ask an AI trained on English data to help you improve such wordings.	Yes, but I still have a suggestion. Even if an AI suggested adding the colon in your revised sentence, I would argue that it is superfluous. Simply write the sentence, in its corrected version, but without the colon (and also add the missing space after the “≥” sign):<br>• The steepest increases in both Ever LD and Current LD prevalence occurred among adolescents aged 12–17 years, females, non-Hispanic Whites, and children from households with college-educated parents or incomes ≥ 200% FPL.<br>• This is a suggestion, not a requirement. For that reason, I wrote “yes” in the “Approved for Publication” column.<br>• The missing space after the “≥” sign must be added, but that is addressed in an earlier row of my feedback.	Yes

No.	Specific Point	Addressed?	Approved for Publication
29	In a nationally representative population from the NSCH, we identified a weighted prevalence of ever-diagnosed LD at 8.80% and current-diagnosed LD at 8.20% among US children aged 6-17 years in 2016-2022. In a nationally representative population from the NSCH, we identified a weighted prevalence of ever-diagnosed LD at 8.80% and current-diagnosed LD at 8.20% among US children who were aged 6–17 years in the period 2016–2022.	Yes, good!	Yes
30	in aged 12-17 years, I literally do not understand what you mean with this wording (here and earlier in the manuscript). Ask an AI why this is hard for readers to understand what you mean. I am sure you mean something meaningful. However, there is probably a language barrier leading to a wording in English that is confusing. The rest of the phrase makes sense but should be reworded (see above).	Yes, but the solution is not grammatically correct.<br>• This is your revised wording: In the subgroups, we also found a significant increase in the average annual change in the prevalence of both ever LD and current LD among those aged 12–17 years, female, non-Hispanic white, individuals with a college education or higher, and those with a family income of 200% FPL or above.<br>• The enumeration contains non-parallel wordings that are ungrammatical in English (even if an AI suggested this version; it is a common mistake, so the AI will likely have such incorrect wordings in its LLM).<br>• Please correct the units of information included in the enumeration so they have a similar structure. For example, you could write it this way: In the subgroups, we also found a significant increase in the average annual change in the prevalence of both ever LD and current LD among those aged 12–17 years, females, non-Hispanic whites, individuals with a college education or higher, and those with a family income of 200% FPL or above.<br>• As you can see, it is only two plural endings I added. However, the enumeration is ungrammatical otherwise.	No

No.	Specific Point	Addressed?	Approved for Publication
31	A study analyzing the association between screen time and developmental disabilities among children aged 0-17 years using NSCH data from 2018-2020, noted a total LD 🚸 Remove the comma here. It is incorrect and confusing. Ask an AI to explain to you WHY the comma is wrong.	Yes, good!	Yes
32	another research conducted by Zablotsky, 🚸 another study conducted by Zablotsky, 🚸 It is grammatically incorrect to write “another research” because “research” is a mass noun (i.e., you cannot count it; like the word ‘milk,’ for example). You cannot say “a” research. Here, too, ask an AI to help you understand what I mean. Writing “Other research” is fine, however, because “other” does not quantify what follows the way “another” does.	Yes, good!	Yes
33	Moreover, it is a lifelong condition 🚸 I highly doubt that the state of the art within theories of learning disabilities agree that learning disabilities are always lifelong conditions. Also, writing “it is a lifelong condition” is weak both from a writing and a thinking standpoint. What EXACTLY do you mean here by “it”? Please provide a more nuanced statement and align it very carefully with leading textbooks / technical literature on learning disabilities. Perhaps learning disabilities are “often” lifelong conditions? At any rate, be more nuanced and follow recent cutting-edge theory in the field.	Yes, much better!	Yes

No.	Specific Point	Addressed?	Approved for Publication
34	Moreover, it is a lifelong condition and older children may have a higher prevalence rate and increasing trend due to longer exposure to the possibility of evaluation and diagnosis.7 There are several problems with this sentence: First, as noted, the claim about "it" being a "lifelong condition" seems implausible and simplistic. Second, your sentence makes THREE major claims, all connected backwards with "Moreover." That is not a good writing style, and it is not argumentatively persuasive to pack all this stuff into one sentence and only have one reference at the end of the sentence. A better approach: Break the claims up. Carefully explain each claim and offer literature support for each claim separately. In other words: First claim: About the long-term nature of (many) learning disabilities [add supporting literature]. Second claim: higher prevalences are being reported for older children [add supporting literature]. Third claim: The higher prevalences being observed for older children may be resulting from ... [explain that idea in detail and then add supporting literature].	Yes, much better!	Yes

No.	Specific Point	Addressed?	Approved for Publication
35	Learning disabilities are difficult to diagnose, because the age at identification depends not only on the type and severity of the learning disabilities, but also on whether there are associated deficits.25 ... This is a very important paragraph for your discussion. What is missing is some sort of advanced organizer after the introductory part of the paragraph explaining the nature of the different observations that then constitute your analysis in the paragraph. After the first two sentences (as of “Nearly all studies ...”), you then discuss gender differences, then ethnic group differences, then SES differences. Prepare the reader for THAT structure before ‘diving into’ those areas. Do you understand what I mean? If you are not sure, show the paragraph and my comment to an AI and ask for help on how to improve the organization of your thoughts here. You might even consider breaking the paragraph up: first paragraph: Main claim and the advanced organizer + second paragraph about the gender differences + third paragraph about ethnic differences + fourth paragraph about the SES differences.	Yes, much better!	Yes
36	Autism Spectrum Disorder (ASD) Check, for example, the APA7 guidelines on writing the names of conditions, disorders, and the like. I’m pretty sure you write them lowercase. Thus: autism spectrum disorder (ASD)	Yes, good!	Yes

No.	Specific Point	Addressed?	Approved for Publication
37	Although the pathogenesis of learning disabilities is currently unclear, I'm skeptical that your blanket statement is accurate. Are you sure this is what state-of-the-art handbooks and the like are currently saying about learning disabilities? I would imagine that the pathogenesis of learning disabilities is not fully understood, but that much is known about at least some of the complex etiological factors that have been implicated in contributing to specific learning disabilities. The term 'learning disabilities' covers a huge range of conditions that themselves have all sorts of definitions. Please re-read a few introductory handbook chapters in the field and reconsider your statement to make sure you are being nuanced in how you make this point.	Yes, excellent solution!	Yes
38	Certain heritable disorders even approach 100% penetrance, What does "penetrance" mean here? Are you sure that is the right word? I'm not sure that is actually a word. Do you mean "penetration"? But if yes, what would that mean?	Yes, and thank you for explaining this word to me. I wasn't familiar with it but now it makes sense! You could also keep the original word, even though I didn't recognize it. Either solution seems fine to me. Remember to add the missing space after ">" in ">95%" (as noted in another row in my feedback for the entire manuscript).	Yes

No.	Specific Point	Addressed?	Approved for Publication
39	Dietary behaviors have also been proposed to play a role in the increases in LD, exemplified by a study that found a high intake of sweetened desserts, fried food, and salt was associated with more learning disability.50 Hm, are you sure this is a strong study? Without knowing the study, my hunch would be that we are talking about comorbidities and that it is really hard to get at causality here. If you cite the study, make sure it is a strong study that actually makes progress towards substantiating a causal link. Is the study an RCT? Was it at least longitudinal? Did they at least use propensity-score matching? Otherwise, I would imagine that parents who allow their children to consume excessive amounts of unhealthy food are also engaging a host of other behaviors that are harmful for their children’s development. Be cautious here. If the study is weak, consider removing or revising your claim.	Thank you for double-checking the study and making the change. It is a very interesting connection, I think. However, for the sake of the scientific focus in your paper, it makes sense to remove the claim based on correlational work here. (an aside: Remember my feedback from further above for writing rebuttals on feedback on peer reviews: It makes it easier for the person checking your responses if you include their wordings verbatim rather than paraphrased. I noticed here, again (and a few other times), that you paraphrased my comments before responding to them. I would avoid doing that in the future for the reason I noted in an earlier response.)	Yes

No.	Specific Point	Addressed?	Approved for Publication
40	associated with learning disability, 👫 associated with learning disabilities, 👫 That is not a trivial language difference in English. I know it looks like a tiny change (from singular to plural), but it is very important that you become aware of these sorts of usage conventions. If you are not sure whether your wording is idiomatic, ask an AI trained on English to help you check your writing. Feel free to share my feedback with an AI so the AI can help you with your language in the manuscript.	Yes, good!	Yes
41	Many proven or potential environmental biological factors 👫 Many substantiated or implicated environmental-biological factors 👫 I would avoid “proven” in this sort of research, because it is generally not possible to truly ‘prove’ these sorts of relationships. At best, we find stronger/better evidence.	Yes, good!	Yes
42	showed that children in the highest quartile of urinary cadmium 👫 “urinary cadmium” what? You probably mean in the highest quartile of cadmium levels in urine samples. If you are taking the term “urinary cadmium” from an article on that topic, say, then the authors have probably established with what they mean by “urinary cadmium” before they start using that shortened form. Check the literature you are citing and be more thorough in your wording here.	Yes, good!	Yes

No.	Specific Point	Addressed?	Approved for Publication
43	had significantly higher odds of LD 👫 had significantly higher odds of having a self-reported LD	Yes, good!	Yes
44	Research has confirmed that learning disabilities are lifelong;[9] 👫 This again sounds very simplistic. I highly doubt that “research” has literally “confirmed” that (all) “learning disabilities”—which is a giant blanket term covering probably hundreds if not thousands of researcher-defined conditions—are “lifelong.” Follow my feedback from above on this point. This is an essential point that must be improved in your writing. Otherwise, your thinking is not nuanced enough for a serious scientific publication. To make sure you are not discouraged by my strong feedback here: Your larger point, which I think is that learning disabilities have long-term, potentially even lifelong consequences for individuals and, therefore, need to be given much attention, is a good and strong point that you definitely can and should make in your article 😊	Yes, good!	Yes
45	with specific learning disability amounted 👫 with a specific learning disability amounted 👫 Here, too: This might seem like a tiny wording change. However, it is a very important chance for learning to write polished English scientific prose. Ask an AI trained on English to help you learn how to catch and fix these sorts of wording issues.	Yes, good!	Yes

No.	Specific Point	Addressed?	Approved for Publication
46	In the US, it's reported that over half of the students receiving special education services do so due to learning disabilities..11 🚸 In the US, over half of students receiving special education services receive them due to learning disabilities.11 🚸 Avoid using contractions in formal scientific papers (it’s 🚸 it is); it goes without saying that this has been reported; moreover, I do wonder about the point you are making conceptually: What does that statistic tell us since I would imagine that access to special educational services normally REQUIRES some sort of diagnosis of a learning disability? Consider addressing that point. My guess is that it is explained in the study you cite. If I am missing your point, then explain your point in the manuscript for thoroughly.	Yes, good!	Yes

No.	Specific Point	Addressed?	Approved for Publication
47	This study has several limitations. First, the cross-sectional design of the NSCH does not permit the identification of how specific factors may influence the diagnosis of LD. Second, the reliance on parental reports for the diagnosis of LD could potentially invite inaccuracies due to misreporting and recall bias. This is your entire discussion of limitations? I find it rather superficial and unconvincing. The authors need to take a serious look at the sample and their method of analyzing the sample and think critically about important limitations and discuss these carefully. I already noted one limitation above about the same. Moreover, you are working with self-reported learning disabilities. Furthermore, you have an extremely undifferentiated operationalization that literally includes all manner of ‘learning disabilities.’ What’s more, you are publishing your research for an international audience but focusing on internal US data. What does that limitation mean for the generalization of your findings beyond the United States? This is a big problem in research in the social sciences including psychology; see, for example, the work on the WEIRD bias by Joseph Henrich.	Yes, great improvement!	Yes

No.	Specific Point	Addressed?	Approved for Publication
48	I am not an expert on learning disabilities, but you authors are, so please put much more effort into creating a well-informed, sincere, and deep discussion of limitations. With a good discussion of limitations, you will greatly improve the scientific value of your work, because science is about building constructively on existing studies. If you fail to provide a thorough and transparent discussion of your study limitations, you are essentially blinding researchers with regard to your work, making it harder for them to build on your work. I have a problem with that. Also, as your limitations section is currently written, researchers will be skeptical when they read your article and thus less likely to cite you. In other words, it is also a matter of establishing trust with your readers by having a detailed and brave limitations section. To be clear: I will not reject a study for being open and thorough about limitations. On the contrary, a deep discussion of limitations is a hallmark of scientific thinking.	Yes, great improvement!	Yes
49	Conclusions: The conclusion is lacking in depth. I agree that you should keep your conclusion focused on concrete key findings and implications and that, therefore, all manner of 'fluff' is to be avoided. However, I think you can do much better here.	Yes, great improvement!	Yes

2. Additional Points in Need of Attention

In addition to the feedback based on my systematic review of how you addressed my original feedback of 29 April 2025, I have a few more points in need of attention based on my review of your revised manuscript. Please also address these points:

50. The abstract contains two undefined abbreviations: “from NSCH” and “with LD” It is generally not best practice to introduce abbreviations without explaining them first. If the abstract length limit allows you to do so, I would write out the terms and then put the abbreviations in parentheses.
51. I noticed an inconsistency in how your reference numbers are placed. Generally, the square brackets with the reference number or numbers follow a space. For example: “and economic performance [6,7].” Sometimes, they are set directly after a word (no space). For example: “identifying learning disabilities[14]”. Please double check the convention/rule used by PLOS One and standardize your format for all of your citation numbers.
52. In some cases, you now have two em-dashes (——) instead of one (—). For example: “and behavioral disorders——such as”. I noted this above, but check the entire manuscript for any remaining instances of “——” and fix them to “—”.
53. between 1997 to 2021, and the National Survey of Children’s Health in 2003 [10–12]. between 1997 to 2021 and the National Survey of Children’s Health in 2003 [10–12]. → The comma needs to be removed. It is incorrect to have a comma in this case.
54. In a clinician reported study, In a clinician-reported study,
55. Your manuscript uses both “healthcare” and “health care.” Both orthographies can be used, I suspect. Please standardize both variants to one. I suggest consistently writing “healthcare” as one word.
56. among children aged between 4 to 15 years among children aged between 4 and 15 years
57. at both national and state levels, and to inspect at both national and state levels and to inspect [better with no comma here]
58. “US” versus “U.S.” versus “United States” Your manuscript is inconsistent in how it uses these three variants. Please standardize all instances of these words. Style guides have different suggestions about this. Most guides advise writing out the name when the country is used as a noun (e.g., “in the United States” and not “in the U.S.”). It is normal to use the abbreviation when the country is being used as an adjective (e.g., “US children” or “U.S. children”). I would suggest always writing out “United States” when it is used as a noun and always writing “U.S.” when it is used as adjective. Here are a few examples of both usages form your manuscript:
    a. in the US [18]. → in the United States [18].
    b. insights from individualistic contexts such as the U.S. and → insights from individualistic contexts such as the United States and”
    c. the population in the US → the population in the United States
    d. among US children who were → among U.S. children who were
    e. in US Children and Adolescents → in U.S. Children and Adolescents
    f. US Office of Management and Budget → U.S. Office of Management and Budget

59. The capitalized words at the start of each requirement should be changed to lower case and an “and” should be added to signal the end of the enumeration: (a) Persistent deficits lasting ≥6 months; (b) Performance substantially below age expectations; (c) Onset during developmental period; (d) Exclusion of alternative explanations (e.g., intellectual disability, inadequate instruction). (a) persistent deficits lasting ≥ 6 months; (b) performance substantially below age expectations; (c) onset during developmental period; and (d) exclusion of alternative explanations (e.g., intellectual disability, inadequate instruction).
60. in institutional settings like orphanages or group homes. in institutional settings such as orphanages or group homes. → This is only a stylistic suggestion, not a required correction. Background: Some writing guides make a distinction between “like” (meaning similar to; for example: “the adults behaved like children”) and “such as” (meaning including or for example). In the noted case in your manuscript, you probably mean settings including orphanages, not settings that are similar to orphanages. However, this is only a suggestion, because not all style guides recognize this difference. Also, many AIs do not pick up on the difference when giving language advice.
61. The funding and oversight for the NSCH are provided by The funding and oversight for the NSCH were provided by
62. Ethical approval for all survey procedures is obtained Ethical approval for all survey procedures was obtained
63. Written consent is obtained through electronic submission or paper mailing, and it is returned to the US Census Bureau [16]. Written consent was obtained through electronic submission or paper mailing, and it was returned to the US Census Bureau [16].
64. a comprehensive representative of the population a comprehensive representation of the population
65. one child under 18 years old one child under 18 years of age
66. interviewing an adult family who interviewing an adult family member who
67. included: (a) Weighted Topical Completion Rate: 27.1%–30.9% (reflecting full questionnaire completion among eligible households); (b) Weighted Interview Completion Rate: 69.7%–78.5% (indicating cooperation among contacted households); (c) Weighted Overall Response Rate: 35.8%–39.1% (representing initial participation). included: (a) the weighted topical completion rate of 27.1%–30.9% (reflecting full questionnaire completion among eligible households); (b) the weighted interview completion rate of 69.7%–78.5% (indicating cooperation among contacted households); and (c) the weighted overall response rate of 35.8%–39.1% (representing initial participation). → Even if in your source these rates are capitalized, it would be better to write them in lower case.
68. asked if a doctor, other health care provider or educator had ever asked whether a doctor, other healthcare provider, or educator had ever [multiple changes; all discussed previously]
69. were subsequently asked if their child were subsequently asked whether their child
70. The outcome variable for this analysis were The outcome variables for this analysis were
71. were “Ever LD” and “Current LD”. were “Ever LD” and “Current LD.” → In formal US English, you place most (but not all!) punctuation marks within the quotation marks, even when the punctuation mark is not actually part of what you are quoting. This is absolutely the rule. I know from my work in Germany that many non-native writers of US English can hardly believe this is actually the rule, because it seems counter-intuitive. It is, however, established practice. The only exceptions are if you are quoting something like a variable name

or a code for software engineering or something similar in which readers might absolutely need to know whether the punctuation mark is actually part of what you are coding. In your case, it is clear that the category name "Current LD" is without the period, even if you include the period before the closing quote. British English does not follow this rule. However, must scientific writing uses US English conventions.

72. The study divided the race/ethnicity into four groups, Hispanic, non-Hispanic white, non-Hispanic black, and other The study divided the race/ethnicity into four groups: Hispanic, non-Hispanic white, non-Hispanic black, and other
73. been diagnosed as having LD, been diagnosed as having an LD,
74. The steepest increases in both Ever LD and Current LD prevalence occurred among: adolescents aged 12–17 years, females, non-Hispanic Whites, and children from households with college-educated parents or incomes ≥200% FPL. The steepest increases in both Ever LD and Current LD prevalence occurred among adolescents aged 12–17 years, females, non-Hispanic Whites, and children from households with college-educated parents or incomes ≥ 200% FPL.
75. likely due to their longer exposure to evaluation and diagnosis possibility [10]. likely due to their longer exposure to evaluation and the possibility of being diagnosed [10]. → The wording "diagnosis possibility" is at bit hard to understand. Is my rewording what you mean?
76. A study using data from NHANES A study using data from the National Health and Nutrition Examination Survey (NHANES) → Always write out abbreviations before you introduce them.
77. Forsyth et al. using data from the National Audit Commission and the Department of Health, reported widespread discrepancy from the median spend/ burden ratio standing at £10,260 per person with learning disability [63]. Using data from the National Audit Commission and the Department of Health, Forsyth et al. reported a widespread discrepancy from the median spend / burden ratio standing at £10,260 per person with a learning disability [63].
78. Suggestion for Fig 1 and 2: The two maps are good enough for publication. However, they are not best practice and their look is probably not what you want for your own publication: the plots are using an unusual projection (the map looks unusually horizontally stretched); the placement of the state abbreviations directly (in a black font) on the colored states is not easily legible; and the rendering of the maps is poor (they are grainy/fuzzy). If you do the plots with ggplot2 in R, you should be able to achieve a much better result. Here is an example of a plot recently done by my wife using ggplot2 for a scientific publication:

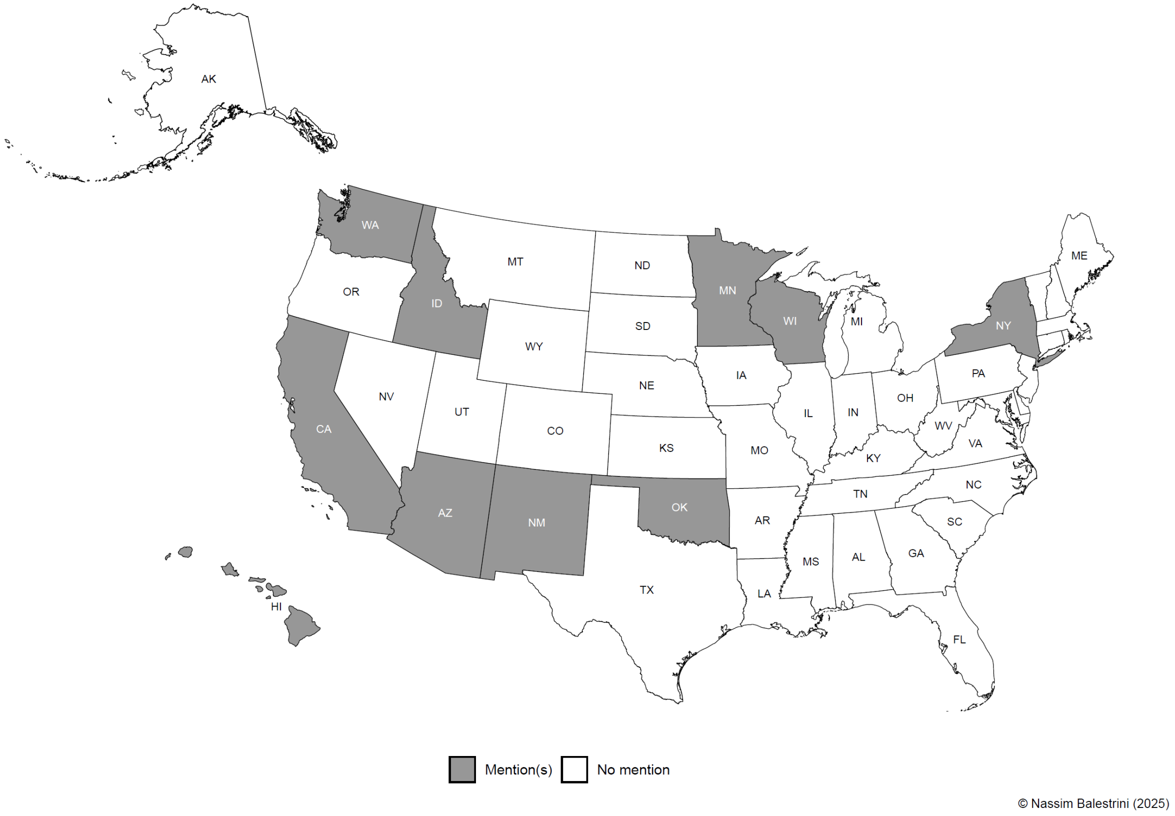

While it is not a heatmap (yours is), the two-letter country abbreviations are easier to read in the states covered in gray because she set the text to appear in white. You could do something similar in your heatmap plots (as of a certain color saturation, the text font switches to white). Also, compare the projection: Her projection is less stretched along the horizontal axis. While no 2-dimensional map projection can be "correct," I believe that the less stretched projection is a more standard solution. If you would like to see the code she used, I would be happy to share it with you (danielpatrickbalestrini@gmail.com).

79. The resolution of Fig 3 is not good, and I can hardly read the individual plot facets. Please try to improve this (also not mandatory, but important). Keep in mind: PLOS One will not help you with your plots. They will simply publish what you give them, even if it is almost or totally illegible. The journal has detailed instructions on how to prepare your plots for publication. Make sure to follow these instructions exactly to avoid a published plot that is of frustratingly poor quality. The more professional your plots look, the more scholars will

take seriously your article. For my PLOS One publication, I used the image-correction website PACE (https://pace.apexcovantage.com/) to prepare the images for the publisher. The result was fine.
