# Supplementary material for: Rising prevalence of parent-reported learning disabilities among U.S. children and adolescents aged 6–17 years: NSCH, 2016–2023
Source: PLoS One. 2025 Oct 22;20(10):e0333850. doi: 10.1371/journal.pone.0333850 (PMC12543174; doi:10.1371/journal.pone.0333850)
Supplement: Attachment — Submitted filename: Comments on PONE-D-24-50967_V3.pdf [file pone.0333850.s005.pdf]

Comments on PONE-D-24-50967, Version 3

I commend the authors on completing an excellent study and also for their willingness to pay attention to the low-level details of scientific writing and manuscript preparation.

I approve of the manuscript's publication now in its current form.

I checked the change-tracked version of the manuscript and the descriptions of changes relating to my feedback. The maps look highly professional, to name just one positive improvement!

I noticed one typographical error and a few tiny things that, ideally, should still be corrected prior to publication, if the authors have the chance:

1. providing a representation representative of the population → providing a comprehensive representation of the population [It looks like the word "comprehensive" was accidentally deleted when changing "representative" to "representation."]
2. The bibliography is set with a different font than the other parts of the manuscript. I don't think this will matter for publication. I suspect the journal will change everything into its standard publication font. However, I'm not sure. If you have the opportunity, you might want to use the standard font you are using in the main manuscript in the bibliography, too.
3. Look at p. 7 of your clean manuscript (and the same spot in the change-tracked version): If you look carefully, you can see that you introduced a smaller font size when pasting in the word "the" throughout the passage, for example at "(a) the weighted" you can see that the "the" you added is in a smaller font. I suspect that the incorrect font size will be automatically corrected when the journal prepares your manuscript. However, I'm not sure. If you have the chance, correct the incorrect font size. If not, it's really okay as it is. Most people will not notice this.
4. In your note for Fig 3, you have the situation in which a full sentence starts with the *p* abbreviation, which you adjusted to lowercase as per my suggestion. It is strange in English to start a sentence with a lowercase letter, which is almost always forbidden. You can leave this as is. If you have the opportunity, you might want to slightly rephrase the wording to avoid the problem. For example, you could write in this one instance: "For trend, *p* was calculated ..." If you are able to change this, check to see whether you have any other sentences that start with "*p* …" (probably not).
5. In your Acknowledgments, you reference the NSCH without writing out the abbreviation. This is correct and consistent in the sense that you did already define the abbreviation in the main article. However, as people often read the Acknowledgments section without reading or before reading the article, it might be a nice service to your readers to slightly rephrase the Acknowledgments section to provide what the abbreviation stands for. Also, it will then match how you refer to the CDC there. In other words: The data used in this study were obtained from the NSCH, ... → The data used in this study were obtained from the National Survey of Children's Health (NSCH), ...

6. You might want to do the same in the Data availability section. However, there, too, it is not incorrect as it is: You already defined the abbreviation, and the Data availability section follows closely at the end of the article.
7. In the Author contributors section, I see four very small optimizations you might want to consider if you have the chance prior to publication (which you may not):
    a. Write authors' initials with spaces between their names: Dr. W.Y. --> Dr. W. Y. [and so on].
    b. Turn the lists of 3+ names into proper enumerations (which are then grammatically/syntactically more polished) and use a simple "and" if two people are named.
    c. Use semicolons to break up the subsections in the second (very long) sentence rather than commas, because, in this case, the commas are confusing due to their use within the enumerations.
    d. Remove the unnecessary capital letters, even if they were stated this way on the PLOS form you had to fill out. This is not a form but a paragraph, so I don't think it is incorrect to use a slightly different capitalization scheme.

    In other words, this is how I would revise the paragraph:

    Dr. W. Y. has full access to all of the data in this study and assumes responsibility for study supervision. Concept and design: W. Y. and Y. Q.; acquisition, analysis, or interpretation of data: W. Y., C. X., Y. L., and H. Y.; drafting of the manuscript: C. X. and Y. L.; critical revision of the manuscript for important intellectual content: all authors; statistical analysis: W. Y., C. X., Y. L., and H. Y.

These are all trivial matters that do not require additional peer review. If the editor would kindly give the authors a chance to make a few more corrections, I think that would be good. It is, however, up to the editor.

To reiterate: The manuscript can, I feel, also be published in its current form. It has fully passed my peer review 😊

Sincerely,
Daniel Balestrini
