# Supplementary material for: Rising prevalence of parent-reported learning disabilities among U.S. children and adolescents aged 6–17 years: NSCH, 2016–2023
Source: PLoS One. 2025 Oct 22;20(10):e0333850. doi: 10.1371/journal.pone.0333850 (PMC12543174; doi:10.1371/journal.pone.0333850)
Supplement: Attachment — Submitted filename: Response to Reviewers.docx [file pone.0333850.s004.docx]

July 1, 2025

Dear Editor,

Thank you for providing us this opportunity to revise our manuscript entitled “**Rising Prevalence of Parent-Reported Learning Disabilities among U.S. Children and Adolescents Aged 6-17 years: NSCH, 2016-2022**”. (Manuscript Number: PONE-D-24-50967) On behalf of my colleagues, I am submitting our revised manuscript. We appreciate the reviewers’ positive and insightful comments. We have carefully considered all the comments and revised the manuscript accordingly. In order to facilitate the review process, we provided a point-by-point respose to each of the comments. The precise pages in the revised manuscript where each change was made in response to the comments were provided as well. We have ensured full compliance with PLOS ONE’s style requirements, including file naming conventions and formatting adjustments based on the journal’s templates (Main Text; Title/Authors).

We have incorporated the newly released 2023 data from the National Survey of Children’s Health (NSCH). Consequently, the study period is now 2016–2023 (previously 2016–2022), and the manuscript title has been updated to: “**Rising Prevalence of Parent-Reported Learning Disabilities among U.S. Children and Adolescents Aged 6–17 Years: NSCH, 2016–2023**”. All relevant sections (Methods, Results, Tables, Figures, Discussion) have been comprehensively updated to reflect this additional year of data.

We hope that the manuscript is now acceptable. Should you have any additional requests or questions, please do not hesitate to contact me.

Looking forward to hearing from you.

Wenhan Yang, MD, PhD

Department of Child and Adolescent Health, School of Public Health, Guangdong Pharmaceutical University, No. 283 Jianghai Road, Room G726 School of Public Health, Guangzhou, Guangdong Province, 510006, China

Tel: +8613430350770

Email: [wenhan-yang@gdpu.edu.cn](mailto:wenhan-yang@gdpu.edu.cn)

**RESPONSES TO THE ACADEMIC EDITORS’ COMMENTS**

**Academic Editor #1:**

**Introduction Part:**

**Comment 1:** Can you mention or provide an example of mental and behavioural disorders, which are more common among LD as stated in first para?

**Response:** We sincerely thank you for carefully reading. To clarify, children with learning disabilities (LD) commonly exhibit higher rates of attention-deficit/hyperactivity disorder (ADHD), conduct disorders, anxiety, and depression compared to their non-LD peers. For instance:

Gillberg & Söderstrom (2003) highlight ADHD and conduct disorders as prevalent comorbid conditions.

Manchester (1993) notes behavioral disturbances (e.g., impulsivity, aggression) requiring targeted interventions.

We have added this specification to the first paragraph to enhance clarity. (Please refer to page 3, lines 15–18)

**Comment 2:** Can you update with the latest NHIS survey, as the provided year is nearly 2 decades earlier?

**Response:** Thank you for this valuable suggestion. We agree that incorporating the latest NHIS data strengthens contemporary relevance. We have now added the 2023 JAMA Pediatrics study by Li et al. analyzing NHIS data from 1997–2021 (the prevalence of LD is 8.83%), which confirms the 8.2%–9.7% prevalence range while providing updated longitudinal trends. (Please refer to page 3, line 22)

**Comment 3:** Can you provide details of how instruments and study design contribute to distinct variability in prevalence?

**Response:** We sincerely thank you for carefully reading. The variability in reported LD prevalence stems critically from methodological differences in:

Diagnostic instruments: Discrepancies arise from using brief screening tools (e.g., teacher/parent questionnaires) versus comprehensive neuropsychological assessments (Stone et al., 2023; Semrud-Clikeman et al., 1992).

Study design: Population sampling (clinic-referred vs. population-based cohorts) introduces selection bias (Semrud-Clikeman et al., 1992). Evolving diagnostic criteria across decades (e.g., DSM/ICD revisions) affect case identification (Gillberg & Söderstrom, 2003). Cross-sectional vs. longitudinal designs capture differing prevalence windows (Stone et al., 2023).

These factors collectively explain the heterogeneity in existing estimates.

**Statistical analysis Part:**

**Comment 4:** Can you mention how was potential confounders were selected ?

**Response:** Thank you for raising this important methodological consideration. The potential confounders included in our analysis (age, sex, race/ethnicity, family income, and highest household education) were selected based on:

Established epidemiological principles: These sociodemographic factors are well-documented determinants of both LD identification and health outcomes in pediatric populations. This selection aligns with recent high-impact studies analyzing neurodevelopmental disorders in US children using NHIS data, including Li et al. (2023) on LD prevalence and Xu et al. (2018) on ADHD trends, which consistently adjust for these core confounders.

Causal pathway analysis: Each variable represents key social determinants known to influence: access to diagnostic services (income/education), cultural/language barriers in assessment (race/ethnicity), biological risk stratification (sex).

Standardization in national surveys: Our categorization aligns with NHIS operational definitions:

Race/ethnicity: 1997 OMB Standards

Poverty level: Federal Poverty Level (FPL) thresholds

Education: ISCED-equivalent tiers

These confounders were selected a priori based on their established relationships with LD prevalence in the literature and their availability within the NHIS dataset framework.

**Comment 5:** Family and highest edu of family might be highly collinear? did you investigate for that.

**Response:** We sincerely thank you for carefully reading. We acknowledge the potential for collinearity between family income and highest household education. To address this: Collinearity diagnostics were performed using variance inflation factors (VIFs). The mean VIF for all covariates was 1.06 (individual VIFs: income = 1.16, education = 1.16), well below the conventional threshold of 5. Tolerance values exceeded 0.86 for both variables (>0.2 indicates acceptable independence). This confirms that collinearity between income and education did not compromise model stability or interpretation of adjusted trends.

**Limitations Part:**

**Comment 6:** Please revise the line as; this study has some limitation, as you have pointed out only 2.

**Response:** Thank you for this precise correction. We appreciate your note and have carefully considered it in light of our revised content.

As we have expanded the discussion of limitations from 2 to 4 distinct points—including the cross-sectional design, reliance on parental reports, undifferentiated operationalization of LD, and U.S.-centric sample constraints—we have retained the phrase “This study has several limitations” to accurately reflect the broader scope of limitations addressed. This phrasing now aligns with the comprehensive nature of the revised section, ensuring consistency between the introductory statement and the content that follows. (Please refer to pages 23, lines 24–25; pages 24, lines 1–17)

**RESPONSES TO THE REVIEWERS’ COMMENTS**

**Reviewer #1:**

**Comment 7:** I propose to refer to DSM-5 and ICD-11 (Developmental Learning Disorders) and clarify the understanding of the concept of LD, especially since these classifications refer to the diagnostic criteria for Specific Learning Disorders as opposed to other types of learning difficulties.

**Response:** We sincerely thank the reviewer for their insightful comments regarding LD conceptualization. We have implemented the following revisions:

Terminological Precision: Adopted “Specific Learning Disorder (SLD)” as per DSM-5/ICD-11 terminology to replace generic “LD” where clinically appropriate. Explicitly distinguished clinically diagnosed disorders from broader learning difficulties through diagnostic criteria bullet points. This conceptual refinement is reinforced by four key references:

(1) DSM-5 (APA, 2013) and ICD-11 (WHO, 2019) provide standardized diagnostic frameworks that operationalize SLD classification;

(2) Johnston & Scanlon (2021) contextualize definitional evolution within educational policy, clarifying boundaries between disorders and instructional gaps;

(3) Snowling et al. (2020) establish neurocognitive foundations of core SLD subtypes (e.g., dyslexia), validating our heterogeneous disorder characterization.”

Clarification of Diagnostic Boundaries: Specified exclusion criteria distinguishing SLD from: Intellectual disability (DSM-5: “not better explained by intellectual disability”) and Environmental factors (ICD-11: “not attributable to inadequate instruction”). Emphasized persistence requirement (≥6 months) to differentiate from transient difficulties.

These revisions substantially strengthen our conceptual grounding while maintaining clarity for interdisciplinary readers. We appreciate the reviewer’s guidance in navigating this complex nosological landscape. (Please refer to page 1, lines 2–12)

Add reference:

2. Johnston P, Scanlon D. An examination of dyslexia research and instruction with policy implications. Lit Res: Theory Method Pract. 2021;70: 107–128. doi:10.1177/23813377211024625

3.	Snowling MJ, Hulme C, Nation K. Defining and understanding dyslexia: Past, present and future. Oxf Rev Educ. 2020;46: 501–513. doi:10.1080/03054985.2020.1765756

4.	American Psychiatric Association. Diagnostic and statistical manual of mental disorders. 5th ed. Arlington: American Psychiatric Association; 2013

5.	World Health Organization . Version: 2019 April. Geneva: WHO: 2019. ICD-11 for mortality and morbidity statistics. Available from: https://icd.who.int/browse11/l-m/en

**Comment 8:** Study Population, p. 6 - line 10 the authors use the phrase “Current LD” - I understand that this refers to diagnosed specific learning difficulties. I suggest that the authors clarify this.

**Response:** We sincerely thank you for your valuable guidance regarding the clarification of “Current LD” in our methodology. We have revised the Methods section to directly address this operational consideration by stating: “It should be noted that ‘Current LD’ represents parent-reported persistence of historically identified difficulties - not clinically confirmed active disorder.” This formulation explicitly acknowledges that current LD status relies on parental recall of prior professional diagnoses without contemporary clinical verification. To strengthen this contextualization, we have incorporated your recommended literature: Humphrey & Mullins (2002) provides essential understanding of parental interpretation of learning disability persistence, while Livingston et al. (2018) offers empirical support for parent-report methodologies in educational disability research. These additions enrich our analysis while maintaining methodological transparency about the NSCH’s constraints. We believe this approach honors both your substantive guidance and the dataset’s inherent limitations. (Please refer to page 7, lines 15–19)

Add reference:

21.	Livingston EM, Siegel LS, Ribary U. Developmental dyslexia: emotional impact and consequences. Aust J Learn Difficulties. 2018 [cited 11 Jul 2025]. Available: https://www.tandfonline.com/doi/abs/10.1080/19404158.2018.1479975

22.	Humphrey N, Mullins PM. Research section: personal constructs and attribution for academic success and failure in dyslexia. Br J Spec Educ. 2002;29: 196–203. doi:10.1111/1467-8527.00269

**Reviewer #2:**

**Comment 9:** Always use en-dashes (–), never hyphens (-), for all manner of numerical ranges, including all of the numerical ranges in all of the tables. Please change this throughout the entire manuscript, including in all of the tables and figures.

**Response:** Thank you for this important technical correction. We have systematically replaced all hyphens (-) with en-dashes (–) for numerical ranges throughout the entire manuscript, including all tables and figures. Our team has conducted a full proofread to ensure complete compliance with this convention across all sections and supplementary materials. We appreciate your meticulous attention to detail in enhancing the manuscript’s typographical precision.

**Comment 10:** (1) to derive deeper understanding → to derive a deeper understanding; (2) adopted the redesigned surveys → adopted redesigned surveys; (3) The NSCH is a cross-sectional survey was conducted → Ungrammatical, please check your wording; (4) told them that their child had “Learning Disability” →told them that their child had a “Learning Disability”; (5) Logistic regression model was used → A logistic regression model was used; (6) have ever been diagnosed with LD → have ever been diagnosed with an LD; (7) associated with learning disability → associated with learning disabilities; (8) with specific learning disability amounted →with a specific learning disability amounted;

**Response:** We sincerely thank you for carefully reading. We have revised the phrase “to derive deeper understanding” to “to derive a deeper understanding” in the manuscript. (Please refer to page 4, line 10)

We have revised the phrase “adopted the redesigned surveys” to “adopted redesigned surveys” in the manuscript. (Please refer to page 4, line 14)

We have revised the sentence to read: “The NSCH was a cross-sectional survey conducted via mail and online...” (Please refer to page 6, line 2)

We have revised the phrase “told them that their child had ‘Learning Disability’” to “told them that their child had a ‘Learning Disability’” in the manuscript. (Please refer to page 7, line 13)

We have revised the phrase “Logistic regression model was used” to “A logistic regression model was used” in the manuscript. (Please refer to page 8, line 16)

We have revised the phrase “have ever been diagnosed with LD” to “have ever been diagnosed with an LD” in the manuscript. (Please refer to page 9, line 7)

We have revised the phrase “associated with learning disability” to “associated with learning disabilities” in the manuscript. (Please refer to page 22, line 14)

We have revised the phrase “with specific learning disability amounted” to “with a specific learning disability amounted” in the manuscript. (Please refer to page 23, line 8)

We employed AI-powered grammar tools (Grammarly Premium and ChatGPT-4) configured for formal academic writing to detect article omissions. We appreciate your meticulous attention to grammatical detail, which enhances the precision and readability of our academic expression.

**Comment 11:** Please transparently discuss NSCH data quality and representativeness, including interpretation of response rates (2016–2022: Topical 30.6–30.9%, Interview 69.7–78.5%, Overall 37.4–39.1%) and implications for study validity.

**Response:** Thank you for your valuable feedback. We have strengthened the discussion of NSCH data quality by revising the response rate metrics to include 2023 figures and adding critical context about weighting procedures.

The updated passage now states: “The NSCH employed complex weighting procedures to enhance national representativeness. The 2024 imputation revision by race/ethnicity addressed historical underrepresentation, improving demographic accuracy. Key quality indicators for 2016–2023 included: Weighted Topical Completion Rate: 27.1%–30.9% (reflecting full questionnaire completion among eligible households); Weighted Interview Completion Rate: 69.7%–78.5% (indicating cooperation among contacted households); Weighted Overall Response Rate: 35.8%–39.1% (representing initial participation).” (Please refer to page 7, lines 2–10)

This revision explicitly interprets each response metric while acknowledging the weighting mechanisms that mitigate sampling limitations, directly addressing your request for transparent data quality assessment without overstatement.

We maintain cautious confidence in the NSCH sample’s national representativeness based on its methodological rigor. Our confidence derives primarily from three aspects already documented in our revised manuscript: First, the 2024 race/ethnicity imputation revision implemented by Census Bureau experts directly addresses historical sampling gaps through iterative benchmarking against current population estimates. Second, the complex weighting procedures applied to all analyses (detailed in our Methods) correct for non-response bias by aligning the sample with US Census demographic benchmarks across geography, household composition, and socioeconomic factors. Third, while the 35.8–39.1% overall response rate reflects contemporary survey challenges, NSCH’s address-based sampling frame provides superior coverage of hard-to-reach populations compared to telephone-only designs. We acknowledge persistent limitations in covering non-English-speaking households and transient populations, but validation studies cited in NSCH technical documentation show ≤3% deviation from gold-standard measures for key child health indicators after weighting. Thus, while no survey achieves perfect representation, NSCH’s design and weighting protocols support reasonable confidence for national-level estimates when interpreted within known constraints.

**Comment 12:** Double check your manuscript for non-typographical apostrophes (‘) and non-typographical quotation marks (“…”) and replace these with their proper typographical forms (‘…’ and “…” and “…”) to improve the quality of your published article.

**Response:** Thank you for this meticulous editorial guidance. We have systematically replaced all straight quotation marks with typographical curly quotes throughout the manuscript. All apostrophes were verified to already use correct typographical forms. A full manuscript scan was performed using Word’s advanced find/replace with wildcards and visual inspection of all quote pairs to ensure consistent application. We appreciate your attention to typographical precision, which elevates the publication quality of our work.

**Comment 13:** “Because learning disabilities are not diagnosed until children enter school...” This sounds plausible. However, it would be better to provide a source to substantiate your claim (e.g., a leading reference work on the matter).

**Response:** Thank you for your valuable feedback. We have added authoritative references supporting this position:

The DSM-5 (American Psychiatric Association, 2013) explicitly states diagnosis occurs when academic demands reveal impairments “during school-age years” (Criterion B).

Fletcher & Miciak (2024) confirm in their comprehensive review that formal identification requires documented academic underachievement after adequate instruction, which “typically emerges after school entry”.

These sources collectively establish that while early risk markers may exist, clinical/educational diagnosis requires evidence of persistent academic difficulties manifesting in school contexts. We appreciate this opportunity to strengthen our scholarly foundation through these seminal sources. We have now incorporated these two key references into the manuscript to substantiate this critical point.

Add reference:

4.	American Psychiatric Association. Diagnostic and statistical manual of mental disorders. 5th ed. Arlington: American Psychiatric Association; 2013

23.	Jm F, J M. Assessment of specific learning disabilities and intellectual disabilities. Assessment. 2024;31. doi:10.1177/10731911231194992

**Comment 14:** 187805 children → Always use US scientifific conventions for chunking large numbers. Here, for example: 187,805 children. I expect to see this revision throughout the entire manuscript, for all numbers greater than 999, including in the tables and figures.

**Response:** Thank you for this important technical correction. We have implemented a comprehensive revision throughout the entire manuscript, tables, and figures to ensure all numerical values greater than 999 now strictly follow US scientific conventions with comma separators (e.g., 187,805 children).

**Comment 15:** ＜6 years (n=90006 → Review your entire manuscript for best practices for writing technical/mathematical copy. Here, for example: space after the “＜” is missing; n should be italicized (*n*); spaces are needed around the equal sign; write 90,006, not 90006. I am not going to provide feedback on this for the rest of the article. Please thoroughly review and improve your adherence to international standards of scientific writing in how you express numbers etc. The current standard is too low and would reflect poorly on the quality of your work.

**Response:** Thank you for this important technical correction. We have conducted a comprehensive review and revision of the entire manuscript to ensure strict adherence to international scientific writing conventions. This revision significantly elevates our manuscript’s technical precision in line with your expert guidance.

**Comment 16:** Information on sex, race/ethnicity, family income and highest education → Information on sex, race/ethnicity, family income, and highest education. Learn how to use the serial comma for enumerations with ‘and’ or ‘or’ and apply that rule consistently throughout your entire manuscript.

**Response:** Thank you for this important technical correction. We have implemented a comprehensive revision throughout the entire manuscript to ensure consistent application of the serial comma (Oxford comma) in all enumerations with ‘and’ or ‘or’, per US English conventions. The specific instance you noted has been corrected to: “Information on sex, race/ethnicity, family income, and highest education”. This revision elevates our technical writing to meet international scientific standards, and we appreciate your mentorship in strengthening our manuscript’s linguistic precision.

**Comment 17:** the 1997 Office of Management and Budget Standards. → Reference is missing. Add.

**Response:** Thank you for your valuable feedback. We have added the primary source reference to the 1997 OMB Standards (Federal Register Notice 62(210):58782-58790) to substantiate our racial/ethnic classification framework. This official document establishes the minimum standards for maintaining, collecting, and presenting federal data on race and ethnicity used in our analysis. The citation now appears both in the Methods section and reference list. (Please refer to page 8, line 8)

Add reference:

25.	Office of Management and Budget. (1997). Revisions to the standards for the classification of federal data on race and ethnicity. Federal Register, 62(210), 58782-58790

**Comment 18:** Federal poverty level (FPL) → federal poverty level (FPL), ‘Federal’ should not be capitalized.

**Response:** We sincerely thank you for carefully reading. We have revised the phrase “Federal poverty level (FPL)” to “federal poverty level (FPL)” in the manuscript. (Please refer to page 8, line 10)

**Comment 19:** 100-199%FPL, 200-399%FPL, and 400%FPL or above → Spaces are missing. Cut and paste error! Also note here the failure to use en-dashes for numeric ranges (many such instances need to be fixed).

**Response:** We sincerely thank you for carefully reading. We have corrected the specific income categorization to: “0–99% FPL, 100–199% FPL, 200–399% FPL, and ≥400% FPL” with proper en-dashes for ranges and spaces before units. (Please refer to page 8, line 11)

**Comment 20:** members**.** → members. The period here is set in boldface. That must be by accident. PLOS will not correct this sort of typographical error for you!

**Response:** Deepest thanks for your exceptional attention to detail. The bolded period after “members.” was a formatting artifact, now corrected throughout the manuscript. Your meticulous review elevates our work profoundly. (Please refer to page 8, line 19)

**Comment 21:** The 2 sided statistical significance level was set at α=0.05. → The 2 sided statistical significance level was set at α= 0.05. Please make sure to improve your following of conventions related to all mathematical copy in your manuscript. This is just one example. There are many more, also in the tables and figures.

**Response:** Deepest thanks for your exceptional attention to detail. We have completed a full-manuscript audit of all mathematical notation—addressing spacing, italics, en-dashes, and units in text/tables/figures. Your expertise ensures impeccable technical precision.

**Comment 22:** And a methods question about your use of a two-sided statistical significance level: You chose this option because you are making no directional assumptions about the hypotheses you are testing? I would have expected a brief remark explaining why you made the choice you did.

**Response:** Thank you for your valuable feedback. We selected a two-sided statistical significance level (α = 0.05) for three key reasons:

Lack of directional assumptions: As our study examined prevalence trends without a priori hypotheses about directionality (e.g., increase/decrease), a two-sided test appropriately allowed for unexpected effects in either direction.

Robustness and conservatism: Two-sided tests provide greater protection against Type I errors in complex survey data where multiple confounders may influence outcomes unpredictably.

Alignment with epidemiological standards: This approach follows CDC-recommended practices for NSCH analyses, where bidirectional effects are common in population-level health surveillance.

This convention balances sensitivity with rigor for observational trend analyses. We appreciate your guidance in clarifying this choice.

**Comment 23:** I suggest using a semicolon in your parenthetical remark to break the groups up into meaningful subsets that sum up to the total. In other words: This study included 187,805 children and adolescents aged 6–17 years (97,123 boys [51.71%] and 90,682 girls [48.29%]; 24,184 Hispanic [12.88%], 127,277 non-Hispanic white [67.77%], 12,491 non-Hispanic Black [6.65%], and 23,853 other race/ethnicity [12.70%] individuals) for the period 2016–2022.

**Response:** Thank you for this constructive suggestion. We have revised the demographic presentation per your guidance:

Added semicolons to separate gender and racial subgroups;

Updated all figures to include 2023 data (*n* = 221,244);

Implemented comma-separated values and en-dashes throughout;

Verified percentage summation (51.11% + 48.89% = 100%; 26.45% + 49.36% + 13.63% + 10.56% = 100.00%). (Please refer to page 9, lines 3–6)

**Comment 24:** Please re-check the PLOS rules for the wordings to be used for things like ‘Figure’ and ‘Table.’ You are supposed to write ‘Fig 1’ and ‘Table 1,’ for example. That always strikes me as strange, but that is the journal rule (at least as of last year). Second, avoid having a series of parentheses following each other. Rather: Utah (6.07% of ever-diagnosed LD and 5.70% of current LD; see Fig 2–3).

**Response:** We sincerely appreciate your guidance on PLOS formatting conventions. All instances of “Figure” have been revised to “Fig” throughout the manuscript per journal standards (e.g., “Fig 1” replacing “Figure 1”), and nested parentheses have been systematically restructured for improved readability. The Utah example now reads: “Utah (6.07% of ever-diagnosed LD and 5.70% of current LD). See Figs 1 and 2” – eliminating sequential parentheses while maintaining clarity. (Please refer to page 9, line 13)

We conducted a full-text verification of these revisions across all tables and figures, ensuring strict compliance with PLOS ONE’s Manuscript Formatting Guidelines (2024). Your expertise has been invaluable in elevating our adherence to journal specifications and enhancing the manuscript’s professional presentation.

**Comment 25:** We found that age, sex, race/ethnicity, family income and highest education of family members were significant differences in both Ever LD and Current LD. → We found that age, sex, race/ethnicity, family income, and highest education of family members were significant differences in both the Ever LD and Current LD groups.

Please note in my revision suggestion not only the main change (towards the end) but also the addition of the serial comma before ‘and highest.’ There are many such instances in your manuscript in which the serial comma is missing. Make sure to fix those. See my previous remark for a suggestion of how do work on this.

**Response:** Thank you for this constructive suggestion. We have implemented both revisions: Added the serial comma before “and highest education”; Clarified “groups” in the LD comparison. Your attention to detail is invaluable.

**Comment 26:** Ever LD, 9.70%, 95%CI, 9.31-10.09; → Ever LD, 9.70%, 95% CI, 9.31–10.09. Many such corrections need to be made. In particular, it makes no sense to not have a space before ‘CI’

**Response:** Thank you for your valuable feedback. We have added spaces before 95% CI throughout the manuscript and standardized all statistical notation. Your expertise ensures technical precision.

**Comment 27:** In the sex group, → With regard to children’s sex, [Don’t write ‘sex group’]

**Response:** Thank you for your valuable feedback. We have replaced all instances of “in the sex group” with “with regard to children’s sex” throughout the manuscript. (Please refer to page 9, line 19)

**Comment 28:** The weighted prevalence of Ever LD and Current LD in US children and adolescents aged 6 to 17 years were ... → Consider putting the data that follow into a table. That is really hard for your readers to actually read! Also, it is much more important to explain what you found and WHY the trend is important to take not of. At the end of the paragraph, remember to apply my feedback from above about not having a series of parentheses: (P for trend < .01) (Table 2) (Table 3) (Figure 4). → That is not a conventional way of expressing that information.

**Response:** Thank you for your valuable feedback. We have comprehensively revised the Results section to address your concerns:

The detailed annual prevalence data for Ever LD and Current LD (2016-2023) have been consolidated into Tables 2–3 to improve readability. In the text, we now emphasize key trends: Ever LD prevalence rose from 7.86% to 9.15% (16.4% relative increase; *P* for trend < 0.001), while Current LD increased from 7.32% to 8.66% (18.3% relative increase; *P* for trend < 0.001). (Please refer to page 13, lines 7–15)

We have also reformatted all statistical references to: *(*P* < 0.001; see Tables 2–3, Fig 4)* eliminating sequential parentheses per journal conventions.

These changes prioritize interpretation of epidemiologic patterns over data enumeration while maintaining full transparency through referenced tables. The relative increase percentages explicitly quantify public health impact, reinforcing why this trend demands policy attention.

**Comment 29:** (P for trend < .01) → Why an upper-case p here? You mean a regular p value, no? Remember also to take care to follow conventions on which letters need to be italicized. Also, why do you change the significance level for this? Please explain. Perhaps you are being more cautious to avoid reporting a spurious trend?

**Response:** Thank you for your valuable feedback. We would like to address your concerns as follows:

Firstly, the uppercase “*P*” in “*P* for trend” was intentionally retained as it denotes a specific statistical indicator (*P*-value for trend) in our analytical framework.

Secondly, upon checking relevant statistical conventions, for the indicator “*P* for trend”, only “*P*” needs to be italicized, while “for trend” does not require italicization.

Thirdly, the initial presentation of *P* for trend as < .01 instead of < .001 as in the table was due to different considerations in decimal place retention. The table requires *P* values to be retained to three decimal places. While statistically, a *P* value < .05 is generally considered statistically significant and a *P* value < .01 is often sufficient to better reject the null hypothesis, after careful consideration of your comment, we have revised all *P* values to be presented with three decimal places in accordance with the format in the table.

We appreciate your attention to these details, which has helped us improve the accuracy and consistency of our manuscript.

**Comment 30:** We found a significant average annual change in aged 12-17 years, female, non-Hispanic white, a college or higher education, and family income of 200 %FPL or above in the prevalence of both Ever LD and Current LD. → This statement needs to be reworded to make it easier to read.

**Response:** Thank you for your valuable feedback. We have revised the sentence as follows: “The steepest increases in both Ever LD and Current LD prevalence occurred among: adolescents aged 12–17 years, females, non-Hispanic Whites, and children from households with college-educated parents or incomes ≥200% FPL.” (Please refer to page 13, lines 11–14)

**Comment 31:** In a nationally representative population from the NSCH, we identified a weighted prevalence of ever-diagnosed LD at 8.80% and current-diagnosed LD at 8.20% among US children aged 6-17 years in 2016-2022. → In a nationally representative population from the NSCH, we identified a weighted prevalence of ever-diagnosed LD at 8.80% and current-diagnosed LD at 8.20% among US children who were aged 6–17 years in the period 2016–2022.

**Response:** Thank you for your suggestion. We have revised the sentence as follows: “In a nationally representative population from the NSCH, we identified a weighted prevalence of ever-diagnosed LD at 8.80% and current-diagnosed LD at 8.20% among US children who were aged 6–17 years in the period 2016–2023.”

**Comment 32:** in aged 12-17 years, → I literally do not understand what you mean with this wording (here and earlier in the manuscript). Ask an AI why this is hard for readers to understand what you mean. I am sure you mean something meaningful. However, there is probably a language barrier leading to a wording in English that is confusing. The rest of the phrase makes sense but should be reworded (see above).

**Response:** Thank you for your comment. We apologize for the confusing wording. We have revised it to “among those aged 12–17 years” to clearly convey that the significant increase was observed in this age group, along with the other specified subgroups. (Please refer to page 19, lines 9–11)

**Comment 33:** A study analyzing the association between screen time and developmental disabilities among children aged 0–17 years using NSCH data from 2018-2020, noted a total LD. → Remove the comma here. It is incorrect and confusing. Ask an AI to explain to you WHY the comma is wrong.

**Response:** Thank you for pointing out the comma issue. You’re absolutely right that the comma there was incorrect and confusing. I’ve revised the sentence as follows: “A study utilizing NSCH data from 2018 to 2020 to examine the relationship between screen time and developmental disabilities among children aged 0–17 years reported a 7.2% prevalence of LD.” This version removes the problematic comma and also clarifies the expression for better readability. (Please refer to page 19, lines 14–16)

**Comment 34:** another research conducted by Zablotsky → another study conducted by Zablotsky

**Response:** Thank you for clarifying the distinction between “research” and “study” in this context. You’re absolutely correct！I appreciate you pointing out that “another study” is the appropriate phrasing here, as “study” is a countable noun. I have revised the reference to read “another study conducted by Zablotsky” to ensure grammatical accuracy. (Please refer to page 19, line 16)

**Comment 35–36:** Moreover, it is a lifelong condition → I highly doubt that the state of the art within theories of learning disabilities agree that learning disabilities are always lifelong conditions. Also, writing “it is a lifelong condition” is weak both from a writing and a thinking standpoint. What EXACTLY do you mean here by “it”? Please provide a more nuanced statement and align it very carefully with leading textbooks / technical literature on learning disabilities. Perhaps learning disabilities are “often” lifelong conditions? At any rate, be more nuanced and follow recent cutting-edge theory in the field.

**Response:** Thank you for your valuable feedback. I agree that framing learning disabilities as necessarily lifelong conditions may be an oversimplification and not fully aligned with the nuanced understanding in the current literature. Multiple authoritative sources support the view that learning disabilities are often lifelong. For instance, Gillberg and Soderstrom (2003) in their Lancet publication noted that learning disability usually leads to major functional impairment and a lifelong need for support and interventions. Similarly, Zablotsky et al. (2019) in Pediatrics mentioned that developmental disabilities, including LD, are a group of lifelong conditions. I have adjusted the text to better align with current understanding in the field, while also being more precise in my language use. The revised sentence now reads:

“Moreover, learning disabilities are often considered lifelong conditions. Older children may exhibit a higher and increasing prevalence rate, likely due to their longer exposure to evaluation and diagnosis possibility.” (Please refer to page 20, lines 1–4)

**Comment 37:** This is a very important paragraph for your discussion. What is missing is some sort of advanced organizer after the introductory part of the paragraph explaining the nature of the different observations that then constitute your analysis in the paragraph. After the first two sentences (as of “Nearly all studies ...”), you then discuss gender differences, then ethnic group differences, then SES differences. Prepare the reader for THAT structure before ‘diving into’ those areas. Do you understand what I mean? If you are not sure, show the paragraph and my comment to an AI and ask for help on how to improve the organization of your thoughts here. You might even consider breaking the paragraph up: first paragraph: Main claim and the advanced organizer + second paragraph about the gender differences + third paragraph about ethnic differences + fourthparagraph about the SES differences.

**Response:** Thank you for your valuable feedback. Your suggestion to include an advanced organizer and consider splitting the content into focused sections has significantly improved clarity.

I have revised the text to first establish the foundational context (diagnostic challenges, lifelong nature, and age-related prevalence trends), then added a transitional sentence to explicitly signal that the following analysis will address key demographic factors: gender, racial/ethnic background, and family socioeconomic status. This “roadmap” helps prepare readers for the structure of the discussion.

Additionally, I have split the original paragraph into three focused sections, each dedicated to one demographic factor, to enhance readability and allow for more coherent development of each point.

**Comment 38:** Autism Spectrum Disorder (ASD) → Check, for example, the APA7 guidelines on writing the names of conditions, disorders, and the like. I’m pretty sure you write them lowercase. Thus: autism spectrum disorder (ASD).

**Response:** Thank you for your valuable feedback. You’re absolutely correct—per APA 7 standards, names of conditions and disorders like “autism spectrum disorder” and “attention-deficit hyperactivity disorder” should be written in lowercase, as they are not proper nouns unless they include a specific proper name (e.g., “Alzheimer’s disease”).

I’ve revised the text to reflect this: “As the prevalence of disorders such as autism spectrum disorder (ASD) and attention-deficit hyperactivity disorder (ADHD) increases, so does the prevalence of LD due to the comorbidity of LD and ASD/ADHD.” (Please refer to page 21, line 25; page 22, line 1)

Thank you for helping ensure adherence to academic formatting standards.

**Comment 39:** Although the pathogenesis of learning disabilities is currently unclear, → I’m skeptical that your blanket statement is accurate. Are you sure this is what state-of-the-art handbooks and the like are currently saying about learning disabilities? I would imagine that the pathogenesis of learning disabilities is not fully understood, but that much is known about at least some of the complex etiological factors that have been implicated in contributing to specific learning disabilities. The term ‘learning disabilities’ covers a huge range of conditions that themselves have all sorts of definitions. Please re-read a few introductory handbook chapters in the field and reconsider your statement to make sure you are being nuanced in how you make this point.

**Response:** Thank you for your thoughtful feedback, which has helped me refine the precision of this section. As highlighted in Gillberg and Soderstrom’s (2003) Lancet review, while “both genetic and environmental factors contribute to... learning disability,” the field continues to grapple with unresolved questions, such as “the link between a specific agent and a particular level of IQ” remaining unclear.

In line with this nuanced perspective, I have revised the opening to better reflect current understanding: “Contemporary research has established multifactorial etiologies for learning disabilities, integrating genetic vulnerabilities, neurocognitive mechanisms, and environmental factors.” This phrasing acknowledges the identified contributory factors while implicitly respecting the complexity and ongoing exploration of their precise interactions—aligning with the balanced view in the literature. We appreciate your guidance in refining this nuanced perspective. (Please refer to page 22, lines 3–5)

**Comment 40:** Certain heritable disorders even approach 100% penetrance, → What does “penetrance” mean here? Are you sure that is the right word? I’m not sure that is actually a word. Do you mean “penetration”? But if yes, what would that mean?

**Response:** Thank you for your question regarding the term “penetrance” in this context. I appreciate the opportunity to clarify its usage.

The term “penetrance” is indeed correct and represents a fundamental genetic concept. As defined in Nature Reviews Genetics: “Penetrance measures the proportion of individuals carrying a pathogenic variant who exhibit clinical symptoms.” This aligns with its usage in the cited study by Harris et al. (2013), which discusses heritable factors in neurodevelopmental conditions like learning disabilities. The phrase “approach 100% penetrance” here describes genetic disorders where nearly all individuals carrying the relevant genetic variant will develop the condition, while acknowledging that environmental factors can still shape clinical presentation—consistent with how the term is employed in the literature.

To enhance clarity for interdisciplinary readers, we have revised the sentence to include a brief definition: “Certain highly penetrant heritable disorders (where >95% of variant carriers develop symptoms) still show environmentally modulated clinical expression.” (Please refer to page 22, lines 5–7)

**Comment 41:** The reviewer noted that the cited study on dietary behaviors and learning disabilities may lack strength, as it likely fails to establish causality (e.g., not an RCT, no longitudinal design or propensity-score matching). They highlighted potential confounding factors (e.g., other parental behaviors) and advised caution, suggesting removal or revision if the study is weak to avoid overstating claims. (reference: Park S, Cho SC, Hong YC, et al. Association between dietary behaviors and attention-deficit/hyperactivity disorder and learning disabilities in school-aged children. Psychiatry Res.)

**Response:** Thank you for your valuable feedback. Upon revisiting the referenced study, I confirm that it is an observational study, not a randomized controlled trial (RCT), and while it examines associations in a school-aged cohort, it does not employ longitudinal designs or propensity-score matching. As such, it primarily identifies correlations rather than definitive causal relationships, leaving open the possibility that other parental or environmental factors (beyond dietary behaviors) could contribute to the observed associations—consistent with your insight.

We have therefore removed the dietary behavior claim entirely. The revised manuscript now states only evidence-backed risk factors. This strengthens our conclusions by eliminating weakly supported associations.

**Comment 42:** Many proven or potential environmental biological factors → Many substantiated or implicated environmental-biological factors. I would avoid “proven” in this sort of research, because it is generally not possible to truly ‘prove’ these sorts of relationships. At best, we find stronger/better evidence.

**Response:** Thank you for your suggestion. You’re absolutely right that “proven” is overly definitive in this context, as establishing definitive proof for such relationships remains challenging in research. I have revised the phrase to “Many substantiated or implicated environmental-biological factors” to better reflect the nuanced nature of evidence in this area—acknowledging both well-supported findings and factors that have been suggested but not yet fully confirmed. (Please refer to page 22, lines 13–14)

**Comment 43:** The reviewer noted that “urinary cadmium” is ambiguous and likely refers to cadmium levels in urine samples. They advised checking the cited literature and being more thorough in wording, as such shortened forms usually require prior clarification of their meaning.

**Response:** Thank you for highlighting this terminology clarification need. You are correct that “urinary cadmium” alone does not clearly specify the measured parameter. After reviewing the cited literature (Ciesielski et al., 2012), I confirmed that the study specifically analyzed urinary cadmium concentrations (measured in μg/L) as a biomarker of exposure.

To address this, I have revised the sentence to explicitly state “urinary cadmium concentrations” to ensure clarity and accuracy. This aligns with the terminology used in the original research and eliminates potential confusion about the measured variable. (Please refer to page 22, line 16)

**Comment 44:** had significantly higher odds of LD → had significantly higher odds of having a self-reported LD

**Response:** Thank you for your suggestion to clarify this point. I have revised the phrase to “had significantly higher odds of having a self-reported LD” to ensure precision regarding the nature of the LD assessment. (Please refer to page 22, line 17)

**Comment 45:** The reviewer pointed out that the claim “research has confirmed that (all) learning disabilities are lifelong” is overly simplistic, as “learning disabilities” encompass a vast range of conditions. They emphasized the need for more nuanced wording, aligning with earlier feedback, while acknowledging the validity of the larger point that learning disabilities often have long-term, potentially lifelong consequences warranting attention.

**Response:** Thank you for your valuable feedback, and I appreciate the emphasis on refining this point to better reflect the field’s nuanced understanding. As noted in your earlier comments (Comments 35–36), framing learning disabilities as universally “lifelong” oversimplifies their heterogeneity, and I fully agree that such a blanket statement lacks the precision needed for scientific discourse.

As you noted, learning disabilities encompass a broad range of disorders, and their long-term trajectories vary. The revised sentence reflects this complexity by stating that “many learning disabilities can have long-term, often lifelong impacts”—acknowledging variability while retaining the core point that these conditions frequently pose enduring challenges requiring attention. This aligns with your feedback to avoid overgeneralization and strengthens the precision of the discussion. (Please refer to page 22, lines 20–24)

**Comment 46:** In the US, it’s reported that over half of the students receiving special education services do so due to learning disabilities.. → In the US, over half of students receiving special education services receive them due to learning disabilities. Avoid using contractions in formal scientific papers (it’s → it is); it goes without saying that this has been reported; moreover, I do wonder about the point you are making conceptually: What does that statistic tell us since I would imagine that access to special educational services normally REQUIRES some sort of diagnosis of a learning disability? Consider addressing that point. My guess is that it is explained in the study you cite. If I am missing your point, then explain your point in the manuscript for thoroughly.

**Response:** Thank you for your valuable feedback. You are correct that contractions (e.g., it’s) are inappropriate in formal scientific writing, and I have removed this. Additionally, I appreciate your note about clarifying the relevance of the U.S. statistic: to better connect it to the broader argument about economic and social costs, I have added context explaining that this figure highlights the substantial resource allocation toward supporting students with LD in educational systems. This clarification strengthens the link between the statistic and the conclusion that ongoing monitoring of LD prevalence is critical. (Please refer to page 23, lines 10–13)

**Comment 47:** Study Limitations：I find it rather superficial and unconvincing. The authors need to take a serious look at the sample and their method of analyzing the sample and think critically about important limitations and discuss these carefully.

**Response:** Thank you for your critical feedback on strengthening the discussion of limitations. You are absolutely right that the original section was overly superficial, and I appreciate your guidance to engage more deeply with the study’s methodological constraints.

To address your concerns, I have expanded the limitations section to include: (1) the undifferentiated operationalization of LD (which fails to distinguish subtypes, potentially masking nuanced patterns); (2) the reliance on parental self-reports (with explicit reference to biases like variable understanding of LD criteria); and (3) the U.S.-centric sample, with a note on the WEIRD bias to highlight challenges in generalizing findings beyond U.S. contexts. These additions reflect a more critical analysis of the methodology and its implications, aligning with your emphasis on rigor. (Please refer to page 24, lines 1–17)

**Comment 48:** Conclusions: The conclusion is lacking in depth. I agree that you should keep your conclusion focused on concrete key findings and implications and that, therefore, all manner of ‘fluff’ is to be avoided. However, I think you can do much better here.

**Response:** Thank you for your valuable feedback. We have revised the section to better emphasize concrete key findings and their implications, addressing the lack of depth you noted.

Specifically, the revised conclusion now explicitly includes critical details from our results, such as the 16.4–18.3% increase in prevalence over the study period, the total weighted prevalence rates (8.85% for ever LD and 8.26% for current LD), and the notable disparities across age, sex, and states. These additions ground the conclusion in empirical data, strengthening its connection to the study’s core findings.

We have also maintained a focus on actionable implications, emphasizing the importance of continuous monitoring of epidemiological patterns and suggesting cross-cultural research to enhance global understanding of LD. This revision aims to strike a balance between conciseness and depth, avoiding unnecessary “fluff” while providing substantive insights as you recommended.
