# Supplementary material for: Rising prevalence of parent-reported learning disabilities among U.S. children and adolescents aged 6–17 years: NSCH, 2016–2023
Source: PLoS One. 2025 Oct 22;20(10):e0333850. doi: 10.1371/journal.pone.0333850 (PMC12543174; doi:10.1371/journal.pone.0333850)
Supplement: Attachment — Submitted filename: Response to Reviewers (Second rework).docx [file pone.0333850.s006.docx]

September 1, 2025

Dear Editor,

Thank you for providing us this opportunity to revise our manuscript entitled “**Rising Prevalence of Parent-Reported Learning Disabilities among U.S. Children and Adolescents Aged 6–17 years: NSCH, 2016-2023**”. (Manuscript Number: PONE-D-24-50967) On behalf of my colleagues, I am submitting our revised manuscript. We appreciate the reviewers’ positive and insightful comments. We have carefully considered all the comments and revised the manuscript accordingly. In order to facilitate the review process, we provided a point-by-point response to each of the comments. The precise pages in the revised manuscript where each change was made in response to the comments were provided as well. We have ensured full compliance with PLOS ONE’s style requirements, including file naming conventions and formatting adjustments based on the journal’s templates (Main Text; Title/Authors).

We hope that the manuscript is now acceptable. Should you have any additional requests or questions, please do not hesitate to contact me.

Looking forward to hearing from you.

Wenhan Yang, MD, PhD

Department of Child and Adolescent Health, School of Public Health, Guangdong Pharmaceutical University, No. 283 Jianghai Road, Room G726 School of Public Health, Guangzhou, Guangdong Province, 510006, China

Tel: +8613430350770

Email: [wenhan-yang@gdpu.edu.cn](mailto:wenhan-yang@gdpu.edu.cn)

**RESPONSES TO THE EDITOR’ S COMMENTS**

**About the changes to the references:**

We sincerely apologize for the oversight in our previous rebuttal letter, where we omitted to detail the changes made to the reference list. We have provided a point-by-point summary of these important updates below.

1. We have replaced the citation to “Lyon, G. R. (*Learning disabilities*, 1996)” with two more recent and comprehensive studies by “Johnston P (*An examination of dyslexia research and instruction with policy implications*, 2021 [2])” and “Snowling MJ (*Defining and understanding dyslexia: Past, present and future*, 2020 [3])” to better support our argument. (Please refer to page 3, line 5)
2. We have added a definition of Learning Disability (LD) in the manuscript and supported it with two new relevant references– “American Psychiatric Association (*Diagnostic and statistical manual of mental disorders. 5th ed*, 2013 [4])” and “World Health Organization (*ICD-11 for mortality and morbidity statistics*, 2019 [5])”, which we had inadvertently overlooked in the previous version. (Please refer to page 3, line 7)
3. We have replaced the reference to “Liu (*Association between serum selenium concentrations and learning disability in a nationally representative sample of U.S. children*, 2022)” with the more recent and comprehensive work by “Li et al. (*Prevalence and Trends in Diagnosed Learning Disability Among US Children and Adolescents From 1997 to 2021*, 2023 [12])”, which provides updated nationwide prevalence trends for learning disability. This change ensures our manuscript is supported by the latest epidemiological evidence. (Please refer to page 3, line 22–23)
4. Upon re-evaluation, we have determined that the reference to “Deere (*Myopia and later physical activity in adolescence: a prospective study*, 2009)” was not directly relevant to the point being made. It has therefore been removed from the manuscript. To correctly cite the primary data source, we have replaced it with the direct report from the US Census Bureau (2022 [18]). (Please refer to page 6, line 6)
5. In response to the reviewer’s feedback, we have added a new paragraph to the Methodssection to clarify the definitional nuances and a potential limitation of the “Current LD” metric. We note that this measure relies on parent-reported persistence of past diagnoses rather than representing a clinically confirmed active condition. To support this clarification, we have incorporated two new references (cited as [21] and [22] in the revised manuscript). Reference [21] (Livingston et al., 2018) exemplifies the surveillance approach underpinning such parent-reported data, while reference [22] (Humphrey & Mullins, 2002) provides scholarly discourse on the potential for historical diagnoses to be conflated with ongoing impairment. We believe this addition enhances the transparency and scholarly foundation of our work. (Please refer to page 7, line 15–19)
6. Upon re-evaluation, we have determined that the reference to “Coviello (*Effects of early nutrition and growth on brain volumes, white matter microstructure, and neurodevelopmental outcome in preterm newborns*, 2018)” was not directly relevant to the point being made. It has therefore been removed from the manuscript.
7. We have added citations to support the assertion regarding the school-age diagnosis of learning disabilities. The statement is now supported by the foundational diagnostic guidelines (American Psychiatric Association, 2013 [4]) and contemporary literature on assessment (Frazier & Mohammed, 2024 [23]). (Please refer to page 7, line 20)
8. To clarify the methodological basis for our classification of race/ethnicity, we have added a citation to the primary source: the 1997 Office of Management and Budget (OMB) standards [25]. This citation now supports our statement that the four groups (Hispanic, non-Hispanic white, non-Hispanic black, and other) were defined according to these established federal guidelines. (Please refer to page 8, line 8)
9. To provide authoritative support for the statement that “learning disabilities are often considered lifelong conditions,” we have supplemented it with a citation to the seminal review article by Gillberg & Soderstrom (2003) [8], which thoroughly discusses the persistent nature of these disorders. (Please refer to page 20, line 1–2)
10. We have added a statement on the generalizability concerns related to our U.S. sample, citing the concept of WEIRD bias from Henrich et al. (2010) [65]. (Please refer to page 24, line 14)

**RESPONSES TO THE REVIEWERS’ COMMENTS**

**Reviewer #2:**

We sincerely thank the reviewer for your thorough and constructive feedback on our manuscript. We greatly appreciate the time and effort dedicated to improving the quality of our work.

In response to the comments provided in this second round of review, we have carefully addressed: (a) all remaining issues from the feedback of 29 April (Section 1), particularly those marked “Approved for Publication: NO,” in a point-by-point manner; and (b) each of the additional problems noted in the revised manuscript (Section 2), also responding point by point.

All changes have been incorporated into the manuscript, and specific responses to each comment are provided below. We believe these revisions have significantly strengthened the manuscript and are grateful for the opportunity to further improve our work.

| **No. Specific Point** | **Response** |
| --- | --- |
| 1 Always use en-dashes (–), never hyphens (-), for all manner of numerical ranges, including all of the numerical ranges in all of the tables. Please change this throughout the entire manuscript, including in all of the tables and figures. | Thank you for this important reminder. We have now systematically replaced all hyphens with en-dashes throughout the entire manuscript, including all tables and figures. Every numerical range has been triple-checked and corrected to ensure strict adherence to typographical standards. We appreciate your meticulous attention to detail, which has significantly improved the professionalism of our manuscript.   - disorders——such as **→** disorders—such as (Please refer to page 3, line 16) - difficulties——not clinically **→** difficulties—not clinically (Please refer to page 7, line 17) - Rao-Scott chi-square tests **→** Rao–Scott chi-square tests (Please refer to page 8, line 15) - 2 sided statistical significance level **→** 2-sided statistical significance level (Please refer to page 8, line 21) - Fig 1. Weighted Prevalence Estimates of Parent-Reported Ever Learning Disability (LD) among Children Aged 6–17 Years by State——United States, 2016–2023 **→** Fig 1. Weighted Prevalence Estimates of Parent-Reported Ever Learning Disability (LD) among Children Aged 6–17 Years by State (United States, 2016–2023) (Please refer to page 12) - Fig 2. Weighted Prevalence Estimates of Parent-Reported Current Learning Disability (LD) among Children Aged 6–17 Years by State——United States, 2016–2023 **→** Fig 1. Weighted Prevalence Estimates of Parent-Reported Current Learning Disability (LD) among Children Aged 6–17 Years by State (United States, 2016–2023) (Please refer to page 12) |
| 6 Double check your manuscript for non-typographical apostrophes (') and non-typographical quotation marks ("…") and replace these with their proper typographical forms (' ' and "…" “…”) to improve the quality of your published article. PLOS will not fix these for you. They will publish what you submit 1:1. | We sincerely thank you for carefully reading. We have revised the phrase “a family's level of education” to “a family’s level of education” in the manuscript. (Please refer to page 22, line 24)  We have thoroughly checked the entire manuscript, including all tables and figures, and have replaced all non-typographical apostrophes and quotation marks with their correct typographical form. |
| 10 Much improved, but not fully revised. Please fix all such remaining problems. For example:   - lasting ≥6 months; → lasting ≥ 6 months; - children <6 years → children < 6 years - and ≥400% FPL → and ≥ 400% FPL - p values in all three tables: for example: <0.001 → < 0.001 - P for trend <0.001 → P for trend < 0.001 [multiple instances] - ≥200% FPL. → ≥ 200% FPL. - (<1.00: P = 0.777; …**→** (< 1.00: P = 0.777; … - 0.001; ≥4.00: P < 0.001). → There is a space missing after “≥”. Also, is a P missing there, too? - where >95% → where > 95% - I’m pretty sure there are a few more such instances. Check the various mathematical relationship signs to make sure you don’t miss any needed corrections of this sort. | Thank you for highlighting this formatting issue. We have carefully checked the entire manuscript, including all tables and figures, and added spaces after all instances of mathematical operators such as “<” and “≥” to ensure consistency with typographical standards.   - lasting ≥6 months; **→** lasting ≥ 6 months; (Please refer to page 3, line 9) - children <6 years **→** children < 6 years (Please refer to page 7, line 21) - and ≥400% FPL **→** and ≥ 400% FPL (Please refer to page 8, line 11; page 10, table 1; page 15, table 2; page 17, table 3.) - We have corrected the formatting of all *p* values in the three tables by adding a space after the “<” symbol. - *P* for trend <0.001 **→** *p* for trend < 0.001 (Please refer to page 13, line 9–11) - ≥200% FPL. **→** ≥ 200% FPL. (Please refer to page 13, line 14) - (<1.00: P = 0.777; …**→** (< 1.00: *p* = 0.777; … (Please refer to page 18, last line) - ≥4.00: *P* < 0.001). **→** ≥ 4.00: *p* < 0.001). (Please refer to page 18, last line) We have checked the manuscript and confirmed that no *p* is missing from that section. A total of four *p* values are present, and we have double-checked their formatting for clarity: (< 1.00: *p* = 0.777; 1.00–1.99: *p* = 0.479; 2.00–3.99: *p* < 0.001; ≥ 4.00: *p* < 0.001). - where >95% **→** where > 95% (Please refer to page 22, line 5–6)   . |
| 11 Much improved, but not fully revised. Please fix all such remaining problems:   - were asked if a doctor, other health care provider or educator had → were asked whether a doctor, other healthcare provider, or educator had [the change of “if” to“whether” and health care to “healthcare” are bonus corrections] - on ever diagnosis information (*n* = 803), current diagnosis information (*n* = 256) and highest parental education (*n* = 875). → on ever-diagnosis information (*n* = 803), current-diagnosis information (*n* = 256), and highest parental education (*n* = 875). [the two added hyphens are bonus stylistic suggestions (not mandatory) for recommended hyphenated adjectival compounds] - prevalence on families, schools and the healthcare system → prevalence on families, schools, and the healthcare system | Thank you for these precise corrections. We have implemented all suggested changes:   - Replaced “if” with “whether” (Please refer to page 7, line 12) - Revised “health care” to “healthcare” (Please refer to page 7, line 12) - Added hyphens in “ever-diagnosis” and “current-diagnosis” (Please refer to page 7, line 22) - Inserted the serial comma before “and” and “or” in the list (Please refer to page 7, line 12; page 8, line 1; page 15, line 3; page 17, line 3; page 23, line 3)   We appreciate your bonus stylistic suggestions, which have further improved the clarity and consistency of our manuscript. |
| 17 Something must have gone wrong either when I copied my feedback into the online review form or when you worked with it. You must write “The 2-sided statistical significance level …” (with a hyphen). The hyphen is missing is my wording as noted in your rebuttal letter, even though I know I typed a hyphen. Please fix this in the manuscript. If you are unsure what I mean, please recheck the PDF version of my feedback of 29 April | Thank you for this correction. We have added the hyphen to “2-sided”in the manuscript (now “The 2-sided statistical significance level...”) and verified that all similar compound adjectives are properly hyphenated throughout the text. We appreciate your meticulous attention to detail. (Please refer to page 8, line 21) |
| 21 Not exactly, but this is okay. At the bottom of page 30, you still have adjacent sets of parentheses: The weighted prevalence of Ever LD rose from 7.86% (95% *CI*, 7.25–8.46) to 9.15% (95% *CI*, 8.55–9.75) (*P* for trend <0.001), while Current LD increased from 7.32% (95% *CI*, 6.73–7.91) to 8.66% (95% *CI*, 8.06–9.25) (*P* for trend <0.001), → I understand why you didn’t want to collapse the information in the two sets of adjacent parentheses into one set in this case. However, there are other ways of avoiding the double sets of adjacent parentheses that would also provide for correctly parsed information. Find another solution, if possible. | Thank you for this astute suggestion. We have eliminated the adjacent parentheses by integrating the trend test results into the second confidence interval parentheses using semicolons. The revised structure now reads:“7.86% (95% *CI*, 7.25–8.46) to 9.15% (95% *CI*, 8.55–9.75; *p* for trend < 0.001)…7.32% (95% *CI*, 6.73–7.91) to 8.66% (95% *CI*, 8.06–9.25; *p* for trend < 0.001).” (Please refer to page 13, line 10–12) |
| 22 Much improved, but not fully revised. Please fix all such remaining problems with the missing serial comma. See the specific observations I added in the line on Point 11. | Thank you for your meticulous attention to grammatical details. We have thoroughly reviewed the entire manuscript and added all missing serial commas before “and” and “or” in enumerations, as specifically highlighted in Point 11. This includes consistent application of the serial comma rule in lists throughout the text, tables, and figure captions. We appreciate your guidance in enhancing the clarity and precision of our writing. (Please see point 11) |
| 26 (P for trend < .01) → Why an upper-case p here? You mean a regular p value, no? Remember also to take care to follow conventions on which letters need to be italicized. Also, why do you change the significance level for this? Please explain. Perhaps you are being more cautious to avoid reporting a spurious trend? | We sincerely apologize for the oversight and misunderstanding in our previous response. Thank you for your patience and clear guidance. You are absolutely correct. Upon carefully reviewing the Publication Manual of the American Psychological Association (7th Edition), we confirm that the convention is indeed to use a lowercase, italicized *p* for *p* values (Section 6.11).  We have now made the following comprehensive corrections throughout the entire manuscript, including all tables, figures, and supplemental materials:   - Changed all instances of “P” to a lowercase, italicized *p*. - Ensured that only the letter p is italicized, not accompanying words (e.g., *p* for trend). - Removed the hyphen from “p-value” to form “p value” in accordance with APA style.   We appreciate your expertise and meticulous review, which has been crucial in helping us adhere to the highest standards of scientific reporting. |
| 28 Yes, but I still have a suggestion. Even if an AI suggested adding the colon in your revised sentence, I would argue that it is superfluous. Simply write the sentence, in its corrected version, but without the colon (and also add the missing space after the “≥” sign):   - The steepest increases in both Ever LD and Current LD prevalence occurred among adolescents aged 12–17 years, females, non-Hispanic Whites, and children from households with college-educated parents or incomes ≥ 200% FPL. - This is a suggestion, not a requirement. For that reason, I wrote “yes” in the “Approved for Publication” column. - The missing space after the “≥” sign must be added, but that is addressed in an earlier row of my feedback. | We sincerely thank you for carefully reading. We have revised the sentence as recommended:   - Removed the superfluous colon after “among”. (Please refer to page 13, line 13) - Added the missing space after the “≥” sign (now: “≥ 200% FPL”). (Please see point 10) |
| 30 Yes, but the solution is not grammatically correct.   - This is your revised wording: In the subgroups, we also found a significant increase in the average annual change in the prevalence of both ever LD and current LD among those aged 12–17 years, female, non-Hispanic white, individuals with a college education or higher, and those with a family income of 200% FPL or above. - The enumeration contains non-parallel wordings that are ungrammatical in English (even if an AI suggested this version; it is a common mistake, so the AI will likely have such incorrect wordings in its LLM). - Please correct the units of information included in the enumeration so they have a similar structure. For example, you could write it this way: In the subgroups, we also found a significant increase in the average annual change in the prevalence of both ever LD and current LD among those aged 12–17 years, females, non-Hispanic whites, individuals with a college education or higher, and those with a family income of 200% FPL or above. - As you can see, it is only two plural endings I added. However, the enumeration is ungrammatical otherwise. | Thank you very much for catching this error in parallel structure and for providing the clear correction. We sincerely appreciate your guidance, which has improved the grammatical quality of our writing. We have revised the sentence as you suggested:   - Changed “female” to “females”. - Changed “non-Hispanic white” to “non-Hispanic whites”.   The corrected sentence now reads:  “In the subgroups, we also found a significant increase... among those aged 12–17 years, females, non-Hispanic whites, individuals with a college education or higher, and those with a family income of 200% FPL or above.” (Please refer to page 19, line 10)  We have also taken the opportunity to check for and correct similar issues with parallel structure elsewhere in the manuscript. Thank you again for your meticulous review. |
| 50 The abstract contains two undefined abbreviations: “from NSCH” and “with LD” → It is generally not best practice to introduce abbreviations without explaining them first. If the abstract length limit allows you to do so, I would write out the terms and then put the abbreviations in parentheses. | Thank you for this important reminder regarding the use of abbreviations. We agree that abbreviations should be defined upon first use.  In our abstract, we introduced both abbreviations with their full terms in the opening sentence:   - “The prevalence of learning disabilities (LD) among children is a critical public health issue; however, recent LD prevalence estimates among children and adolescents aged 6–17 years, as reported by the National Survey of Children’s Health (NSCH), remain largely unexplored.”   As the abstract has a strict word limit, we followed the standard practice of defining them once at the first mention. However, we sincerely appreciate your vigilance on this matter. If the editor and reviewer feel it would enhance clarity, we would be happy to explore alternative phrasing within the word count constraints. |
| 51 I noticed an inconsistency in how your reference numbers are placed. Generally, the square brackets with the reference number or numbers follow a space. For example: “and economic performance [6,7].” Sometimes, they are set directly after a word (no space). For example: “identifying learning disabilities[14]”. Please double check the convention/rule used by PLOS One and standardize your format for all of your citation numbers. | Thank you for highlighting this formatting inconsistency. We have thoroughly reviewed and standardized all in-text citations throughout the manuscript to strictly adhere to PLOS ONE’s official reference guidelines, which state:  “References are listed at the end of the manuscript and numbered in the order that they appear in the text. In the text, cite the reference number in square brackets (e.g., ‘We used the techniques developed by our colleagues [19] to analyze the data’).”  Specifically, we have added a space before all reference brackets:   - “identifying learning disabilities[14]” → “identifying learning disabilities [14]” (Please refer to page 4, line 8) - “for Specific Learning Disorders[4]” → “for Specific Learning Disorders [4]” (Please refer to page 3, line 7)   We appreciate your meticulous attention to detail, which has strengthened the professionalism of our manuscript. |
| 52 In some cases, you now have two em-dashes (——) instead of one (—). For example: “and behavioral disorders——such as”. I noted this above, but check the entire manuscript for any remaining instances of “——” and fix them to “—”. | Thank you for your meticulous attention to formatting details. We have thoroughly checked the entire manuscript and corrected all instances of double em-dashes (——) to single em-dashes (—).   - “and behavioral disorders——such as” → “and behavioral disorders—such as” (Please refer to page 3, line 16) - “identified difficulties——not clinically” → “identified difficulties—not clinically” (Please refer to page 7, line 17)   We appreciate your meticulous attention to detail, which has strengthened the professionalism of our manuscript. |
| 53 between 1997 to 2021, and the National Survey of Children’s Health in 2003 [10–12]. → between 1997 to 2021 and the National Survey of Children’s Health in 2003 [10–12]. → The comma needs to be removed. It is incorrect to have a comma in this case.  57 at both national and state levels, and to inspect → at both national and state levels and to inspect [better with no comma here] | Thank you for this correction. We have removed the unnecessary comma as instructed:   - “between 1997 to 2021, and the National Survey of Children’s Health in 2003 [10–12].” →“between 1997 to 2021 and the National Survey of Children’s Health in 2003 [10–12].” (Please refer to page 3, line 22) - “at both national and state levels, and to inspect” →“at both national and state levels and to inspect” (Please refer to page 5, line 6)   We have also conducted a full manuscript scan to ensure no similar grammatical errors remain. We appreciate your meticulous review. |
| 54 In a clinician reported study, → In a clinician-reported study | Thank you for this correction. We have revised the phrase as instructed:   - “In a clinician reported study” →“In a clinician-reported study” (Please refer to page 4, line 2)   We have also conducted a full manuscript scan to ensure no similar grammatical errors remain. We appreciate your meticulous review. |
| 55 Your manuscript uses both “healthcare” and “health care.” → Both orthographies can be used, I suspect. Please standardize both variants to one. I suggest consistently writing “healthcare” as one word. | Thank you for this helpful suggestion. We have standardized the term throughout the manuscript by consistently using the closed compound form “healthcare” (as one word) in all instances, replacing any variants of “health care.” We appreciate your attention to detail in improving the consistency of our terminology. |
| 56 among children aged between 4 to 15 years → among children aged between 4 and 15 years | Thank you for this correction. We have revised the phrase as instructed:   - “among children aged between 4 to 15 years” → “among children aged between 4 and 15 years” (Please refer to page 4, line 4)   We have also conducted a full manuscript scan to ensure no similar grammatical errors remain. We appreciate your meticulous review. |
| 58 “US” versus “U.S.” versus “United States” → Your manuscript is inconsistent in how it uses these three variants. Please standardize all instances of these words. Style guides have different suggestions about this. Most guides advise writing out the name when the country is used as a noun (e.g., “in the United States” and not “in the U.S.”). It is normal to use the abbreviation when the country is being used as an adjective (e.g., “US children” or “U.S. children”). I would suggest always writing out “United States” when it is used as a noun and always writing “U.S.” when it is used as adjective. Here are a few examples of both usages form your manuscript:   - a. in the US [18]. → in the United States [18]. - b. insights from individualistic contexts such as the U.S. and → insights from individualistic contexts such as the United States and” - c. the population in the US → the population in the United States - d. among US children who were → among U.S. children who were - e. in US Children and Adolescents → in U.S. Children and Adolescents - f. US Office of Management and Budget → U.S. Office of Management and Budget | Thank you for your clear and practical guidance on standardizing country nomenclature. We have revised the phrase as instructed:   - in the US [18]. → in the United States [18]. (Please refer to page 6, line 6; page 23, line 9) - the population in the US → the population in the United States (Please refer to page 6, line 19) - insights from individualistic contexts such as the U.S. and → insights from individualistic contexts such as the United States and (Please refer to page 25, line 2) - prevalence among US children → prevalence among U.S. children (Please refer to page 2, line 16) - the US Census Bureau → the U.S. Census Bureau (Please refer to page 6, line 15) - in US Children and Adolescents → in U.S. Children and Adolescents (Please refer to page 9, line 2; page 10, line 1; page 13, line 6; page 14, line 1; page 16, line 1) - US Office of Management and Budget → U.S. Office of Management and Budget (Please refer to page 11, line 3) - among US children who were → among U.S. children who were (Please refer to page 19, line 4) - 7.74% among US children → 7.74% among U.S. children (Please refer to page 19, line 18) - of the US population → of the U.S. population (Please refer to page 23, line 17)   All instances have been updated accordingly in the text, tables, and figures. Your suggestion has greatly improved the stylistic consistency of our manuscript, and we sincerely appreciate your help. |
| 59 The capitalized words at the start of each requirement should be changed to lower case and an “and” should be added to signal the end of the enumeration: (a) Persistent deficits lasting ≥6 months; (b) Performance substantially below age expectations; (c) Onset during developmental period; (d) Exclusion of alternative explanations (e.g., intellectual disability, inadequate instruction). → (a) persistent deficits lasting ≥ 6 months; (b) performance substantially below age expectations; (c) onset during developmental period; and (d) exclusion of alternative explanations (e.g., intellectual disability, inadequate instruction). | Thank you for this precise feedback. We have revised the enumeration as suggested:   - Changed all initial capitalized words to lowercase. - Added the conjunction “and” before the final item to properly signal the end of the list.   The corrected text now reads:  “(a) persistent deficits lasting ≥ 6 months; (b) performance substantially below age expectations; (c) onset during developmental period; and (d) exclusion of alternative explanations (e.g., intellectual disability, inadequate instruction).” (Please refer to page 3, line 9–12)  We have also applied this formatting convention to all similar enumerations throughout the manuscript to ensure consistency. We appreciate your attention to detail in enhancing the clarity and grammatical precision of our writing. |
| 60 in institutional settings like orphanages or group homes. → in institutional settings such as orphanages or group homes. → This is only a stylistic suggestion, not a required correction. Background: Some writing guides make a distinction between “like” (meaning similar to; for example: “the adults behaved like children”) and “such as” (meaning including or for example). In the noted case in your manuscript, you probably mean settings including orphanages, not settings that are similar to orphanages. However, this is only a suggestion, because not all style guides recognize this difference. Also, many AIs do not pick up on the difference when giving language advice. | Thank you for this thoughtful stylistic suggestion. We agree that “such as” more precisely conveys the intended meaning of inclusion rather than comparison. We have revised the sentence to: “in institutional settings such as orphanages or group homes.” (Please refer to page 6, line 8)  This change has been applied consistently throughout the manuscript where similar contexts occur. We appreciate your nuanced guidance, which has enhanced the clarity and precision of our writing. |
| 61 The funding and oversight for the NSCH are provided by → The funding and oversight for the NSCH were provided by | Thank you for this correction. We have revised the phrase as instructed:   - “The funding and oversight for the NSCH are provided by” → “The funding and oversight for the NSCH were provided by” (Please refer to page 6, line 11)   We have also conducted a full manuscript scan to ensure no similar grammatical errors remain. We appreciate your meticulous review. |
| 62 Ethical approval for all survey procedures is obtained → Ethical approval for all survey procedures was obtained | Thank you for this correction. We have revised the phrase as instructed:   - “Ethical approval for all survey procedures is obtained” → “Ethical approval for all survey procedures was obtained” (Please refer to page 6, line 13) |
| 63 Written consent is obtained through electronic submission or paper mailing, and it is returned to the US Census Bureau [16]. → Written consent was obtained through electronic submission or paper mailing, and it was returned to the US Census Bureau [16]. | Thank you for this correction. We have revised the sentence as instructed:   - “Written consent is obtained through electronic submission or paper mailing, and it is returned to the US Census Bureau [16].” → “Written consent was obtained through electronic submission or paper mailing, and it was returned to the US Census Bureau [16].” (Please refer to page 6, line 14–15) |
| 64 a comprehensive representative of the population → a comprehensive representation of the population | Thank you for this correction. We have revised the phrase as instructed:   - “a comprehensive representative of the population” → “a comprehensive representation of the population” (Please refer to page 6, line 18) |
| 65 one child under 18 years old → one child under 18 years of age | Thank you for this correction. We have revised the phrase as instructed:   - “one child under 18 years old” → “one child under 18 years of age” (Please refer to page 6, line 22) |
| 66 interviewing an adult family who → interviewing an adult family member who | Thank you for this correction. We have revised the phrase as instructed:   - “interviewing an adult family who” → “interviewing an adult family member who” (Please refer to page 7, line 2) |
| 67 included: (a) Weighted Topical Completion Rate: 27.1%–30.9% (reflecting full questionnaire completion among eligible households); (b) Weighted Interview Completion Rate: 69.7%–78.5% (indicating cooperation among contacted households); (c) Weighted Overall Response Rate: 35.8%–39.1% (representing initial participation). → included: (a) the weighted topical completion rate of 27.1%–30.9% (reflecting full questionnaire completion among eligible households); (b) the weighted interview completion rate of 69.7%–78.5% (indicating cooperation among contacted households); and (c) the weighted overall response rate of 35.8%–39.1% (representing initial participation).  → Even if in your source these rates are capitalized, it would be better to write them in lower case. | Thank you for this correction. We have revised the sentence as instructed:   - “included: (a) Weighted Topical Completion Rate: 27.1%–30.9% (reflecting full questionnaire completion among eligible households); (b) Weighted Interview Completion Rate: 69.7%–78.5% (indicating cooperation among contacted households); (c) Weighted Overall Response Rate: 35.8%–39.1% (representing initial participation).” → “included: (a) the weighted topical completion rate of 27.1%–30.9% (reflecting full questionnaire completion among eligible households); (b) the weighted interview completion rate of 69.7%–78.5% (indicating cooperation among contacted households); and (c) the weighted overall response rate of 35.8%–39.1% (representing initial participation).” (Please refer to page 7, line 6–10)   We appreciate your attention to detail in enhancing the clarity and grammatical precision of our writing. |
| 68 asked if a doctor, other health care provider or educator had ever →asked whether a doctor, other healthcare provider, or educator had ever [multiple changes; all discussed previously] | Thank you for this correction. We have revised the phrase as instructed:   - “asked if a doctor, other health care provider or educator had ever” → “asked whether a doctor, other healthcare provider, or educator had ever” (Please refer to page 7, line 12) |
| 69 were subsequently asked if their child → were subsequently asked whether their child | Thank you for this correction. We have revised the phrase as instructed:   - “were subsequently asked if their child” → “were subsequently asked whether their child” (Please refer to page 7, line 14) |
| 70 The outcome variable for this analysis were → The outcome variables for this analysis were | Thank you for this correction. We have revised the phrase as instructed:   - “The outcome variable for this analysis were” → “The outcome variables for this analysis were” (Please refer to page 8, line 3) |
| 71 were “Ever LD” and “Current LD”. → were “Ever LD” and “Current LD.”  In formal US English, you place most (but not all!) punctuation marks within the quotation marks, even when the punctuation mark is not actually part of what you are quoting. This is absolutely the rule. I know from my work in Germany that many non-native writers of US English can hardly believe this is actually the rule, because it seems counter-intuitive. It is, however, established practice. The only exceptions are if you are quoting something like a variable name or a code for software engineering or something similar in which readers might absolutely need to know whether the punctuation mark is actually part of what you are coding. In your case, it is clear that the category name “Current LD” is without the period, even if you include the period before the closing quote. British English does not follow this rule. However, must scientific writing uses US English conventions. | We are profoundly grateful for this exceptionally clear and pedagogically valuable feedback. We have revised the phrase as instructed:   - were “Ever LD” and “Current LD”. → were “Ever LD” and “Current LD.” (Please refer to page 8, line 3)   Thank you for taking the time to provide such a thoughtful and helpful background explanation. We have carefully moved the period inside the closing quotation marks in the sentence and, as you suggested, have thoroughly checked and corrected all similar instances throughout the manuscript to ensure full consistency with US English typographical standards. |
| 72 The study divided the race/ethnicity into four groups, Hispanic, non-Hispanic white, non-Hispanic black, and other →The study divided the race/ethnicity into four groups: Hispanic, non-Hispanic white, non-Hispanic black, and other | Thank you for this correction. We have revised the phrase as instructed:   - “The study divided the race/ethnicity into four groups, Hispanic, non-Hispanic white, non-Hispanic black, and other” → “The study divided the race/ethnicity into four groups: Hispanic, non-Hispanic white, non-Hispanic black, and other” (Please refer to page 8, line 6) |
| 73 been diagnosed as having LD, →been diagnosed as having an LD, | Thank you for this correction. We have revised the phrase as instructed:   - “been diagnosed as having LD,” → “been diagnosed as having an LD,” (Please refer to page 9, line 9)   We have also conducted a full manuscript scan to ensure no similar grammatical errors remain. We appreciate your meticulous review. |
| 74 The steepest increases in both Ever LD and Current LD prevalence occurred among: adolescents aged 12–17 years, females, non-Hispanic Whites, and children from households with college-educated parents or incomes ≥200% FPL. → The steepest increases in both Ever LD and Current LD prevalence occurred among adolescents aged 12–17 years, females, non-Hispanic Whites, and children from households with college-educated parents or incomes ≥ 200% FPL. | Thank you for this correction. We have revised the sentence as instructed:   - The steepest increases in both Ever LD and Current LD prevalence occurred among: adolescents aged 12–17 years, females, non-Hispanic Whites, and children from households with college-educated parents or incomes ≥200% FPL. → The steepest increases in both Ever LD and Current LD prevalence occurred among adolescents aged 12–17 years, females, non-Hispanic Whites, and children from households with college-educated parents or incomes ≥ 200% FPL. (Please refer to page 14, line 11–14)   We have also conducted a full manuscript scan to ensure no similar grammatical errors remain. We appreciate your meticulous review. |
| 75 likely due to their longer exposure to evaluation and diagnosis possibility [10]. → likely due to their longer exposure to evaluation and the possibility of being diagnosed [10].  → The wording “diagnosis possibility” is at bit hard to understand. Is my rewording what you mean? | Thank you so much for this thoughtful and precise correction. We have revised the phrase as instructed:   - likely due to their longer exposure to evaluation and diagnosis possibility [10]. → likely due to their longer exposure to evaluation and the possibility of being diagnosed [10]. (Please refer to page 21, line 3–4)   Your rewording— “the possibility of being diagnosed” —is exactly what we meant to convey, and it is undoubtedly clearer and more idiomatic than our original phrasing. We appreciate your meticulous review. |
| 76 A study using data from NHANES → A study using data from the National Health and Nutrition Examination Survey (NHANES)  → Always write out abbreviations before you introduce them. | Thank you so much for this crucial correction. We sincerely appreciate you catching this oversight. You are absolutely right to point out that we failed to properly introduce the NHANES abbreviation upon its first use, which is an essential convention for clear scientific writing. We have revised the phrase as instructed:   - A study using data from NHANES → A study using data from the National Health and Nutrition Examination Survey (NHANES) (Please refer to page 23, line 15–16)   Furthermore, we have conducted a thorough check of the entire manuscript to ensure that all other abbreviations are properly defined at their first occurrence. Your meticulous review has significantly improved the clarity and professionalism of our work, and we are truly grateful for your invaluable guidance. |
| 77 Forsyth et al. using data from the National Audit Commission and the Department of Health, reported widespread discrepancy from the median spend/ burden ratio standing at £10,260 per person with learning disability [63]. → Using data from the National Audit Commission and the Department of Health, Forsyth et al. reported a widespread discrepancy from the median spend / burden ratio standing at £10,260 per person with a learning disability [63]. | Thank you for this correction. We have revised the phrase as instructed:   - Forsyth et al. using data from the National Audit Commission and the Department of Health, reported widespread discrepancy from the median spend/ burden ratio standing at £10,260 per person with learning disability [63]. → Using data from the National Audit Commission and the Department of Health, Forsyth et al. reported a widespread discrepancy from the median spend / burden ratio standing at £10,260 per person with a learning disability [63]. (Please refer to page 24, line 5–7) |
| 78 Suggestion for Fig 1 and 2: The two maps are good enough for publication. However, they are not best practice and their look is probably not what you want for your own publication: the plots are using an unusual projection (the map looks unusually horizontally stretched); the placement of the state abbreviations directly (in a black font) on the colored states is not easily legible; and the rendering of the maps is poor (they are grainy/fuzzy). If you do the plots with ggplot2 in R, you should be able to achieve a much better result. | We are immensely grateful for your insightful and constructive feedback on Figures 1 and 2. Your suggestions were exceptionally clear and practical, and they significantly elevated the visual quality and professionalism of our maps.  Specifically, we have implemented all of your recommendations:   - Adjusted the map to use a more appropriate projection to avoid horizontal distortion; - Implemented automatic text color switching to white for better legibility against darker shaded regions, greatly enhancing readability; - Addressed the issue of small, tightly-grouped states by using leader lines for their labels. This method prevents the map from becoming cluttered and ensures that all state abbreviations are clearly legible, even for the smallest states.   Your guidance not only improved these figures but also deepened our understanding of effective data visualization for publication. Thank you for your invaluable help and for contributing so meaningfully to the quality of our manuscript. |
| 79 The resolution of Fig 3 is not good, and I can hardly read the individual plot facets. Please try to improve this (also not mandatory, but important). Keep in mind: PLOS One will not help you with your plots. They will simply publish what you give them, even if it is almost or totally illegible. The journal has detailed instructions on how to prepare your plots for publication. Make sure to follow these instructions exactly to avoid a published plot that is of frustratingly poor quality. The more professional your plots look, the more scholars will take seriously your article. For my PLOS One publication, I used the image-correction website PACE (https://pace.apexcovantage.com/) to prepare the images for the publisher. The result was fine. | We are incredibly grateful for this exceptionally helpful and practical advice. Thank you for taking the time not only to identify the issue with the resolution of Fig 3 but also to provide the specific, solution-oriented recommendation to use the PACE website.  We have followed your suggestion precisely. The figure has been re-exported from OriginPro at a higher resolution and subsequently processed through the PACE tool (https://pace.apexcovantage.com/) to ensure it fully meets PLOS ONE's publication standards for image quality. We are much happier with the new version's clarity and legibility.  We have also taken this opportunity to review all other figures in the manuscript against the journal’s detailed instructions to prevent any similar issues. Your guidance has been invaluable in helping us present our work in the most professional manner possible, and we deeply appreciate your mentorship. |
